# GDE5/Gpcpd1 activity determines phosphatidylcholine composition in skeletal muscle and regulates contractile force in mice

**DOI:** 10.1038/s42003-024-06298-z

**Published:** 2024-05-20

**Authors:** Rahmawati Aisyah, Noriyasu Ohshima, Daiki Watanabe, Yoshiko Nakagawa, Tetsushi Sakuma, Felix Nitschke, Minako Nakamura, Koji Sato, Kaori Nakahata, Chihiro Yokoyama, Charlotte R. Marchioni, Thanutchaporn Kumrungsee, Takahiko Shimizu, Yusuke Sotomaru, Toru Takeo, Naomi Nakagata, Takashi Izumi, Shinji Miura, Berge A. Minassian, Takashi Yamamoto, Masanobu Wada, Noriyuki Yanaka

**Affiliations:** 1 Graduate School of Integrated Sciences for Life, Hiroshima University, Hiroshima, Japan; 2 Graduate School of Medicine, Gunma University, Gunma, Japan; 3 Graduate School of Humanities and Social Sciences, Hiroshima University, Hiroshima, Japan; 4 Graduate School of Sport and Health Sciences, Osaka University of Health and Sport Sciences, Osaka, Japan; 5 Center for Animal Resources and Development (CARD), Kumamoto University, Kumamoto, Japan; 6 Department of Pediatrics, University of Texas Southwestern Medical Center, Dallas, TX USA; 7 Aging Stress Response Research Project Team, National Center for Geriatrics and Gerontology, Aichi, Japan; 8 Natural Science Center for Basic Research and Development, Hiroshima University, Hiroshima, Japan; 9 Faculty of Health Care, Teikyo Heisei University, Tokyo, Japan; 10 Graduate School of Nutritional and Environmental Sciences, University of Shizuoka, Shizuoka, Japan

**Keywords:** Homeostasis, Metabolomics

## Abstract

Glycerophosphocholine (GPC) is an important precursor for intracellular choline supply in phosphatidylcholine (PC) metabolism. GDE5/Gpcpd1 hydrolyzes GPC into choline and glycerol 3-phosphate; this study aimed to elucidate its physiological function in vivo. Heterozygous whole-body GDE5-deficient mice reveal a significant GPC accumulation across tissues, while homozygous whole-body knockout results in embryonic lethality. Skeletal muscle-specific GDE5 deletion (*Gde5* skKO) exhibits reduced passive force and improved fatigue resistance in electrically stimulated gastrocnemius muscles in vivo. GDE5 deficiency also results in higher glycolytic metabolites and glycogen levels, and glycerophospholipids alteration, including reduced levels of phospholipids that bind polyunsaturated fatty acids (PUFAs), such as DHA. Interestingly, this PC fatty acid compositional change is similar to that observed in skeletal muscles of denervated and Duchenne muscular dystrophy mouse models. These are accompanied by decrease of GDE5 expression, suggesting a regulatory role of GDE5 activity for glycerophospholipid profiles. Furthermore, a DHA-rich diet enhances contractile force and lowers fatigue resistance, suggesting a functional relationship between PC fatty acid composition and muscle function. Finally, skinned fiber experiments show that GDE5 loss increases the probability of the ryanodine receptor opening and lowers the maximum Ca^2+^-activated force. Collectively, GDE5 activity plays roles in PC and glucose/glycogen metabolism in skeletal muscle.

## Abstract (web summary)

Skeletal muscle-specific GDE5/Gpcpd1 deletion leads to glycerophosphocholine accumulation, which alters skeletal muscle phospholipid profiles and reduces passive force in electrically stimulated skeletal muscles in vivo.

## Introduction

Choline is an essential nutrient for normal cell functions and occurs mainly in the form of phosphatidylcholine (PC), the major component of membrane phospholipids^1^. Depending on the tissue type, PC synthesis is regulated by two independent pathways: the CDP-choline (Kennedy) pathway and the phosphatidylethanolamine (PE) methylation pathway. In the CDP-choline pathway, PC synthesis from free choline involves choline kinase, phosphocholine cytidylyltransferase, and choline/ethanolamine phosphotransferase. Alternatively, the PE methylation pathway consumes *S*-adenosylmethionine as the major biological methyl donor in a reaction catalyzed by phosphatidylethanolamine *N*-methyltransferase (PEMT)^2^. contrast with progress in understanding PC synthesis pathways, the regulation of the PC hydrolysis remains an incomplete puzzle. PC degradation mainly relies on two pathways: the phospholipase D (PLD) pathway, which catalyzes PC hydrolysis to free choline and phosphatidic acid (PA)^3^, and the phospholipase A_1_/phospholipase A_2_ (PLA_1_/PLA_2_) and lysophospholipase pathway, which hydrolyzes PC into free fatty acids and glycerophosphocholine (GPC)^4^. GPC is widely proposed not only as an intermediate of the PC degradation pathway, but also as one of the major forms of stored choline. However, the enzyme hydrolyzing GPC to choline in vivo remains unknown. In mammals, the glycerophosphodiesterase (GDE/Gpcpd) family of enzymes, which deacylates glycerophospholipids into glycerol phosphate and alcohols, such as free choline, ethanolamine, or inositol, is believed to hydrolyze GPC^5,6^. We previously identified mammalian GDEs and showed that GDE5/Gpcpd1 is the sole cytosolic enzyme highly expressed in skeletal muscle, which effectively hydrolyzes GPC into glycerol 3-phosphate (Gro3P) and choline in vitro (Fig. S1a)^5,7^. Since GPC is a water-soluble intermediate of choline metabolism, GDE5 may be the key to solving the PC-choline turnover puzzle.

Alteration of choline and its metabolites in response to physical activity, environment, and pathological conditions may be the physiological link between choline metabolism and muscle functions^8,9^. In animal studies, a spontaneous recessive mutation within the choline kinase beta (*Chkb*) gene can cause progressive muscular dystrophy, suggesting a physiological significance of total PC biosynthesis during skeletal muscle development^10,11^. Recent studies have focused on the fatty acid composition of membrane PC, widely recognized as the quality of lipids (lipoquality), because phospholipid compositional changes can affect membrane fluidity and signal transduction. Accumulating evidence shows that impaired PC metabolism is closely associated with pathological conditions such as liver failure^12^, Parkinson’s disease^13^, and Alzheimer’s disease^14^. Moreover, recent reports have demonstrated that PC composition in skeletal muscle is significantly altered not only in response to physical exercise and fat supplementation but also under pathological conditions^15–17^. However, the mechanism of endogenous regulation of PC composition and its physiological significance in skeletal muscle functions remain obscure.

In an attempt to solve the choline–PC turnover puzzle, we explored the physiological importance of GDE5, a candidate enzyme in the PC degradation pathway in vivo, in modulating PC and choline metabolism in skeletal muscle. We generated skeletal muscle-specific GDE5-deficient (*Gde5* skKO) mice to investigate the role of GDE5 in a tissue-specific manner. We examined the effect of GDE5 depletion on GPC levels, and its effect on glycolysis-related metabolites and PC composition. Furthermore, we compared GDE5 expression and PC composition in mouse atrophy models to those in *Gde5* skKO mice. The results of this study may lead to a better understanding of the function of GDE5 as a key enzyme for PC composition and skeletal muscle properties.

## Results

### Phenotype analysis of whole-body *Gde5* (*Gpcpd1*) knockout mice

**Fig. 1:**
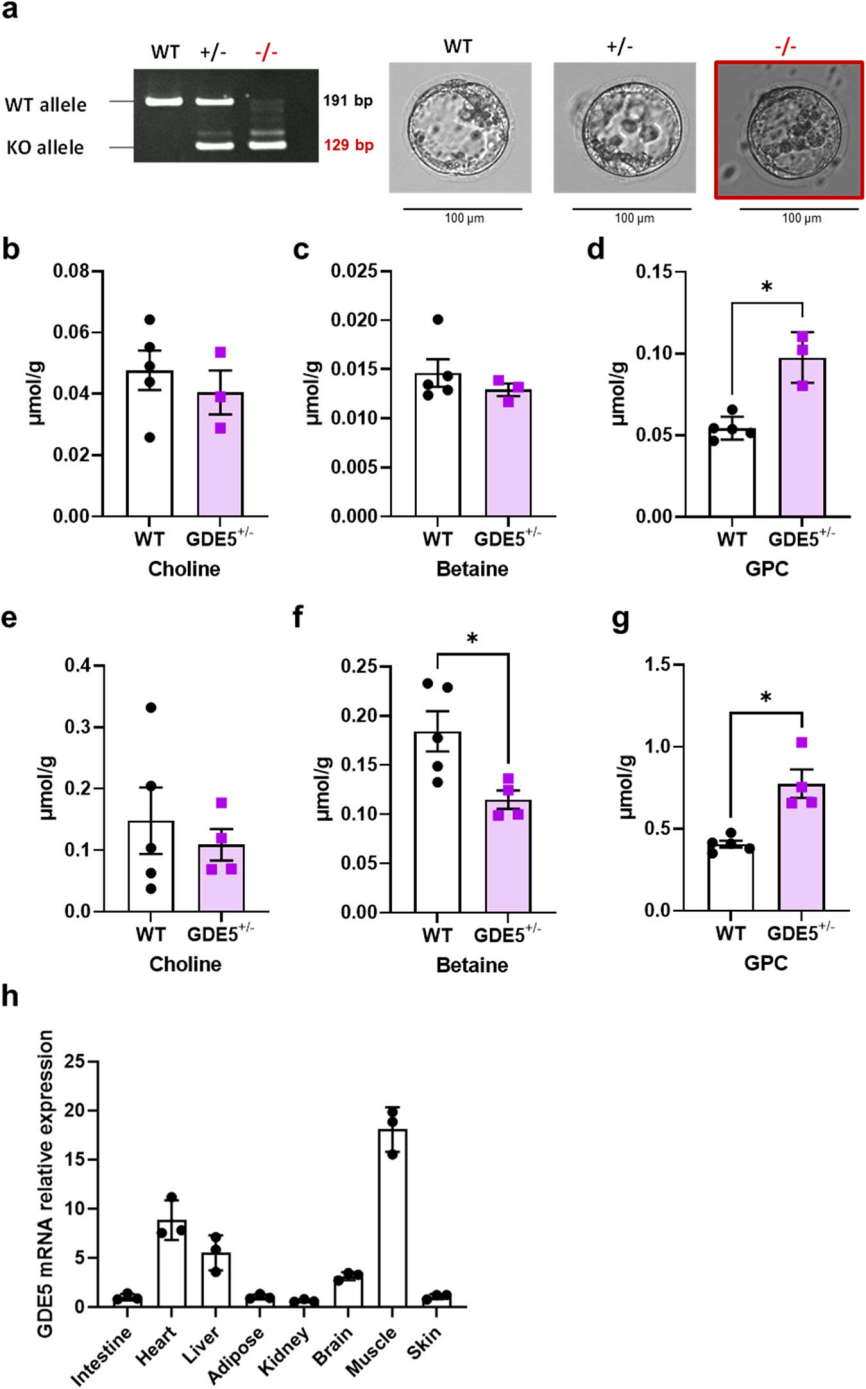
Embryonic lethality of *Gde5*^−/−^ mice and higher GPC level in skeletal muscle and liver of *Gde5*^+/−^ mice. **a** PCR genotyping using DNA from single blastocyst. Isolated WT, *Gde5*^+/−^ and *Gde5*^−/−^ mutant blastocysts. **b**–**d** Choline metabolites levels in skeletal muscle of *Gde5*^*+/−*^ and WT mice fed with standard AIN93 diet. **e**–**g** Choline metabolites levels in livers of *Gde5*^*+/−*^ and WT mice fed with standard AIN93 diet. **h** *Gde5* mRNA expression on various tissues in WT mice. Values are means ± SEM. Statistical analysis was performed with Student’s *t* test. *p < 0.05.

To clarify the role of GPC/choline metabolism, we tried to generate *Gde5* KO mice by using the CRISPR-Cas9 system. The amino acid sequence of GDE5/Gpcpd1 revealed that the GDE5 protein contains a catalytic domain that shares a common motif (amino acids 320–385) with other mammalian GDEs. A guide RNA (gRNA) was encoded in the exon 11 coding region (55 bp), which corresponds to the GDE domain of mouse GDE5^7^. We created whole-body *Gde5* KO mice using an all-in-one CRISPR-Cas9 vector expressing two gRNAs to target both regions outside exon 11 in *Gde5* (Fig. S1b). PCR analysis confirmed *Gde5* allele in genomic DNA isolated from tail biopsies of the offspring. Intercrossing between heterozygote *Gde5*^*+/−*^ parents only produced *Gde5*^*+/−*^ and WT mice but not *Gde5*^*−/−*^ mutant mice. Next, we performed in vitro fertilization with eggs and sperm from *Gde5*^*+/−*^ parents and isolated the *Gde5*^*−/−*^ mutant blastocysts (Fig. 1a), suggesting that GDE5 deficiency does not affect blastocyst development. Because whole-body *Gde5*^*−/−*^ mutation is embryonically lethal, *Gde5*^*+/−*^ mutant mice were further analyzed to determine GPC metabolism in vivo. Body weight and serum metabolite levels were comparable between *Gde5*^*+/−*^ and WT mice (Fig. S2). We measured choline metabolite levels in *Gde5*^*+/−*^ mice fed on a standard AIN93 diet (Fig. 1b–g) and found that the GPC levels increased in skeletal muscles (Fig. 1d) and livers (Fig. 1g), whereas choline levels were not significantly different between *Gde5*^*+/−*^ and WT mice (Fig. 1b, e). Because dietary choline may supply endogenous choline to these tissues, we further investigated whether a choline-deficient diet alters the choline levels in *Gde5*^*+/−*^ and WT mice. We found that the body weight, serum metabolite levels, and tissue choline levels were not significantly different between *Gde5*^*+/−*^ and WT mice (Fig. S2). These suggest that heterozygous GDE5 deletion actually affects the GPC hydrolysis in vivo, although choline metabolism seems to be functioning regardless of choline-deficient diet. Given the wide use of a choline-deficient, high-fat diet (CD-HFD) to induce a mouse model of NASH, we further examined whether CD-HFD alters the choline levels and serum marker levels in *Gde5*^*+/−*^ and WT mice. Our findings revealed no significant differences in the serum metabolite levels and hepatic choline levels between *Gde5*^*+/−*^ and WT mice (Fig. S3). These observations suggest that choline metabolism in *Gde5*^*+/−*^ remains functional possibly through PEMT pathway.

### Contractile function and fatigue in skeletal muscle-specific *Gde5* knockout mice

**Fig. 2:**
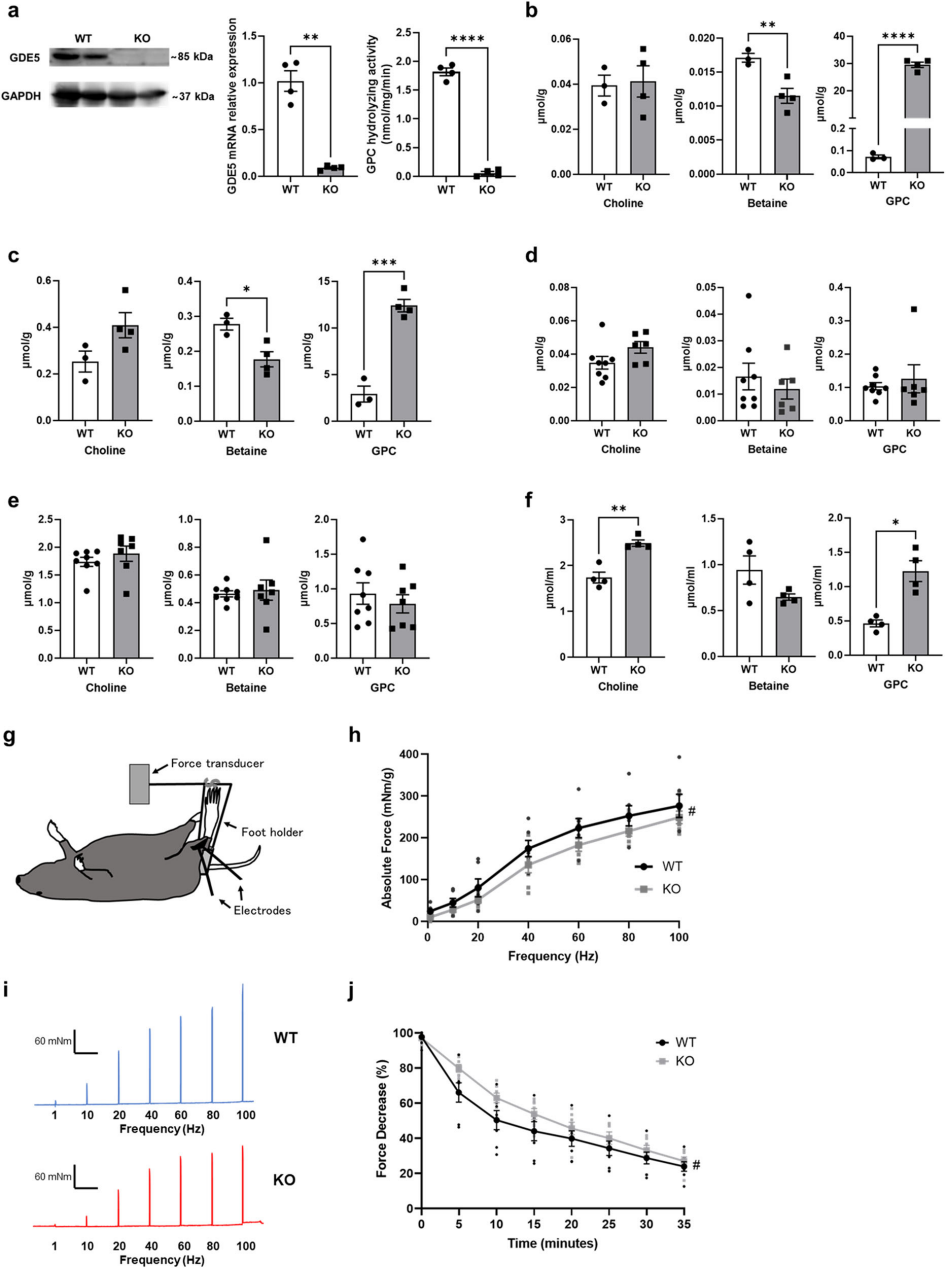
Abnormal GPC/choline metabolism and reduced passive force in the skeletal muscle of *Gde5* skKO mice. **a** Western blot, mRNA expression, and GPC hydrolyzing activity in skeletal muscle of *Gde5* skKO and WT mice. **b**–**f** Choline metabolites levels in skeletal muscle (**b**), liver (**c**), kidney (**d**), white adipose tissue (**e**), and serum (**f**) of *Gde5* skKO and WT mice. **g** Schematic figure of in vivo model for contractile force and fatigability experiment. **h** In vivo contractile force test of *Gde5* skKO and WT mice (*n* = 7). **i** Representative examples of contractile force test. **j** Fatigability test of *Gde5* skKO and WT mice (*n* = 7). Values are means ± SEM. Statistical analysis was performed with Student’s *t* test (**a**–**f**) and two-way ANOVA (**h**, **j**). *p < 0.05; **p < 0.01; ***p < 0.001; ****p < 0.0001; #*p* < 0.05 (main effect).

GDE5 mRNA was highly expressed in mouse skeletal muscle (Fig. 1h). To explore the biological significance of GDE5 expression in skeletal muscle, floxed mice were generated using the CRISPR-Cas9 and PITCh (Precise Integration into Target Chromosome) systems^18^, wherein latter uses a microhomology-mediated end-joining-directed plasmid as a donor (Fig. S4a). The in vitro transcribed gRNA and recombinant Cas9 protein were microinjected into C57BL/6 zygotes, and the surviving zygotes were transferred into pseudopregnant ICR female mice. We confirmed two loxP sites inserted into the introns flanking exon 11 of the *Gde5* gene by nucleotide sequencing (Fig. S4b). Next, we generated skeletal muscle-specific *Gde5* KO mice (*Gde5* skKO mice) using the Cre-loxP system and HSA-Cre transgenic mice for selective expression of the Cre protein in skeletal muscle. We analyzed the GDE5 expression level in *Gde5* skKO skeletal muscles, uncovering a significant decrease in both GDE5 mRNA and protein levels and a considerably lowered GPC hydrolyzing activity in the skeletal muscles (Figs. 2a and S5). No difference was observed in the body weight of *Gde5* skKO and WT mice (Fig. S6a). Among the tissues assessed in this study, only the quadriceps muscle weight was slightly but significantly increased in *Gde5* skKO mice (Fig. S6b-k). mRNA expressions of muscle fiber types were also comparable between the *Gde5* skKO and WT mice (Fig. S6l). To confirm a state of choline metabolism, we measured GPC and metabolite levels in the *Gde5* skKO mice. The GPC level was markedly accumulated in the skeletal muscle of *Gde5* skKO mice, whereas the betaine level was reduced in the tissue (Fig. 2b). Notably, the GPC level was also increased in the liver tissues of *Gde5* skKO mice (Fig. 2c–e). As a significant change in GDE5 expression was not observed within the liver tissue of *Gde5* skKO (Fig. S7a), the increased serum GPC level (Fig. 2f) likely contributed to the increased GPC level in liver cells. These results suggest a close association between the skeletal muscle and liver tissues in GPC/choline metabolism.

Studies have examined alterations in choline and its metabolites in response to physical activity and under pathological conditions to clarify the physiological link between choline metabolism and skeletal muscle functions. Here, we investigated whether contractile force and fatigability were affected by GDE5 deficiency using in vivo stimulation (Fig. 2g). We observed decreased contractile force in the muscles of *Gde5* skKO mice (Fig. 2h, i), while force decline while testing fatigability was less steep compared to the WT mice (Fig. 2j), strongly suggesting that *Gde5* skKO mice experience reduced muscle force but have higher resilience to fatigue.

### GDE5 deficiency alters glucose metabolism

**Fig. 3:**
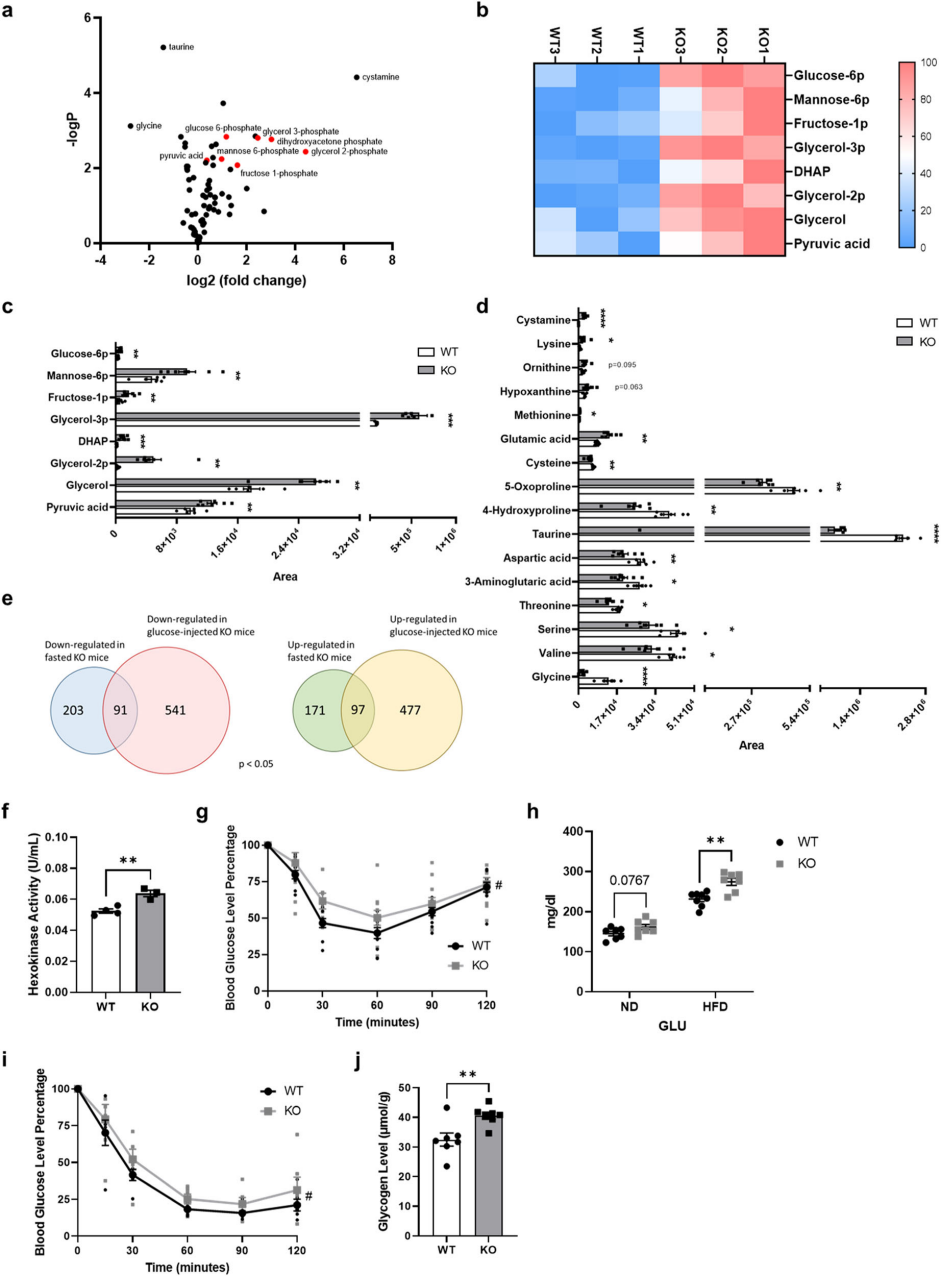
Abnormal glucose metabolism in the skeletal muscle of *Gde5* skKO mice. **a** Volcano plot of water-soluble metabolites in *Gde5* skKO and WT skeletal muscle. **b** Heatmap representation of glucose metabolism-related metabolites levels in *Gde5* skKO and WT skeletal muscle. **c** Glucose metabolism-related metabolites levels in *Gde5* skKO and WT skeletal muscle (*n* = 6–7). **d** Amino acids and their related metabolites levels in *Gde5* skKO and WT skeletal muscle (*n* = 6–7). **e** Venn diagram of DEGs between *Gde5* skKO under fasting and 1 h-post 20% glucose injection. **f** Hexokinase activity of *Gde5* skKO and WT skeletal muscle. **g** Insulin tolerance test of *Gde5* skKO and WT mice under normal chow diet (*n* = 7). **h** Fasting blood glucose levels of *Gde5* skKO and WT mice (*n* = 7) under chow diet and high-fat diet. ND, normal chow diet; HFD, high-fat diet. **i** Insulin tolerance test of *Gde5* skKO and WT mice under high-fat diet (*n* = 7). **j** Glycogen level of *Gde5* skKO and WT skeletal muscle. Values are means ± SEM. Statistical analysis was performed with Student’s *t* test (**c**, **d**, **f**, **h**, **j**) and two-way ANOVA (**g**, **i**). #*p* < 0.05 (main effect); *p < 0.05; **p < 0.01; ***p < 0.001; ****p < 0.0001.

We next investigated whether GPC/choline metabolism interacts with other forms of metabolism in skeletal muscles. As GPC is an important organic osmolyte in mammalian tissues^19^, GPC accumulation in skeletal muscle following GDE5 deficiency may affect other hydrophilic metabolites, which may subsequently alter other modes of metabolism. Here, we assessed the metabolomic profiles of skeletal muscles from *Gde5* skKO and WT mice, and found that 27 of the 85 water-soluble metabolites analyzed differed significantly (Fig. 3a). Interestingly, several glycolysis-related metabolites, including dihydroxyacetone phosphate (DHAP), fructose 1-phosphate, glucose 6-phosphate (G6P), and pyruvic acid, were markedly increased in *Gde5* skKO mice (Fig. 3b–d). Moreover, there was an unexpected, striking increase in Gro3P in the *Gde5* skKO mice, even though Gro3P is a product of GDE5 reaction (Fig. 3c). As a key metabolite involved in the intersection of glucose, lipid, and energy metabolism^20^, Gro3P can also be synthesized from DHAP, which is increased and possibly feeds into the Gro3P pool. Interestingly, glycerol 2-phosphate (β-glycerophosphate) was also increased in the *Gde5* skKO mice. These findings suggest that glucose metabolism is altered in *Gde5* skKO skeletal muscles. Next, we performed DNA microarray analysis and identified 574 upregulated and 632 downregulated genes in the skeletal muscles of *Gde5* skKO mice compared to WT mice 1 h after injecting 20% glucose. Then, we carried out a comparative analysis based on additional transcriptomic data with fasting, which identified 97 upregulated and 91 downregulated genes that overlapped in *Gde5* skKO mice under both conditions (fed and fasted) (Fig. 3e). Further, we observed elevated hexokinase activity (Fig. 3f) in the skeletal muscles of *Gde5* skKO mice, which is consistent with the increased G6P level. *Gde5* skKO mice also showed blunted insulin sensitivity (Fig. 3g). In an attempt to further elucidate the changes in glucose metabolism, we fed a high-fat diet (HFD, 60% of calories from fat) to *Gde5* skKO and WT mice for 20 weeks. We observed elevated fasting blood glucose levels (Fig. 3h) and reduced insulin sensitivity in *Gde5* skKO mice (Fig. 3i) without significant changes in other serum markers (Fig. S7b–e). Notably, no marked difference was observed in GLUT4 protein expression levels within the whole membrane fraction compared to that in the WT skeletal muscles (Fig. S7f, g). Additionally, in *Gde5* skKO mice fed on a normal diet during fasting, a greater glycogen content was observed without any difference in serum insulin levels (Figs. 3j and S7h). Typically, glycogen levels are tightly regulated. Because degradation of muscle glycogen (e.g., by exercise) leads to increased glucose-derived glycogen phosphate^21^, we next measured glycogen C6-phosphate levels in the skeletal muscles of *Gde5* skKO and WT mice. *Gde5* skKO mice showed a 50% decrease in average glycogen C6-phosphate content compared to WT mice, suggesting that the higher glycogen content in the *Gde5* skKO mice could be a consequence of a lower glycogen degradation rate compared to WT mice (Fig. S7i). These findings raise the possibility that choline and glucose/glycogen metabolism are interconnected through the GDE5 enzyme and that alterations in glucose/glycogen metabolism affect the skeletal muscle contractile profile.

Aside from glucose metabolism-related metabolites, metabolomic profiling also revealed altered amino acid and amino acid-related metabolite levels and suggested dysregulated amino acid metabolism in *Gde5* skKO skeletal muscle (Figs. 3d and S7j). Low taurine and hydroxyproline levels have been reported in aging mice^22,23^. Taurine also acts as an organic osmolyte^22^, suggesting that maintaining adequate levels of taurine is important in preserving osmotic balance, particularly during GPC accumulation. In addition, reduced serine and glycine levels reportedly impair muscle regeneration^24^, whereas increased glutamic acid was observed in an animal model of Duchenne muscular dystrophy (mdx mice)^25^. Hence, these data indicate impaired muscle function in response to *Gde5* skKO.

### GDE5 deficiency shifts phospholipid composition to n-6 rich species and similar change occurs in atrophy models

**Fig. 4:**
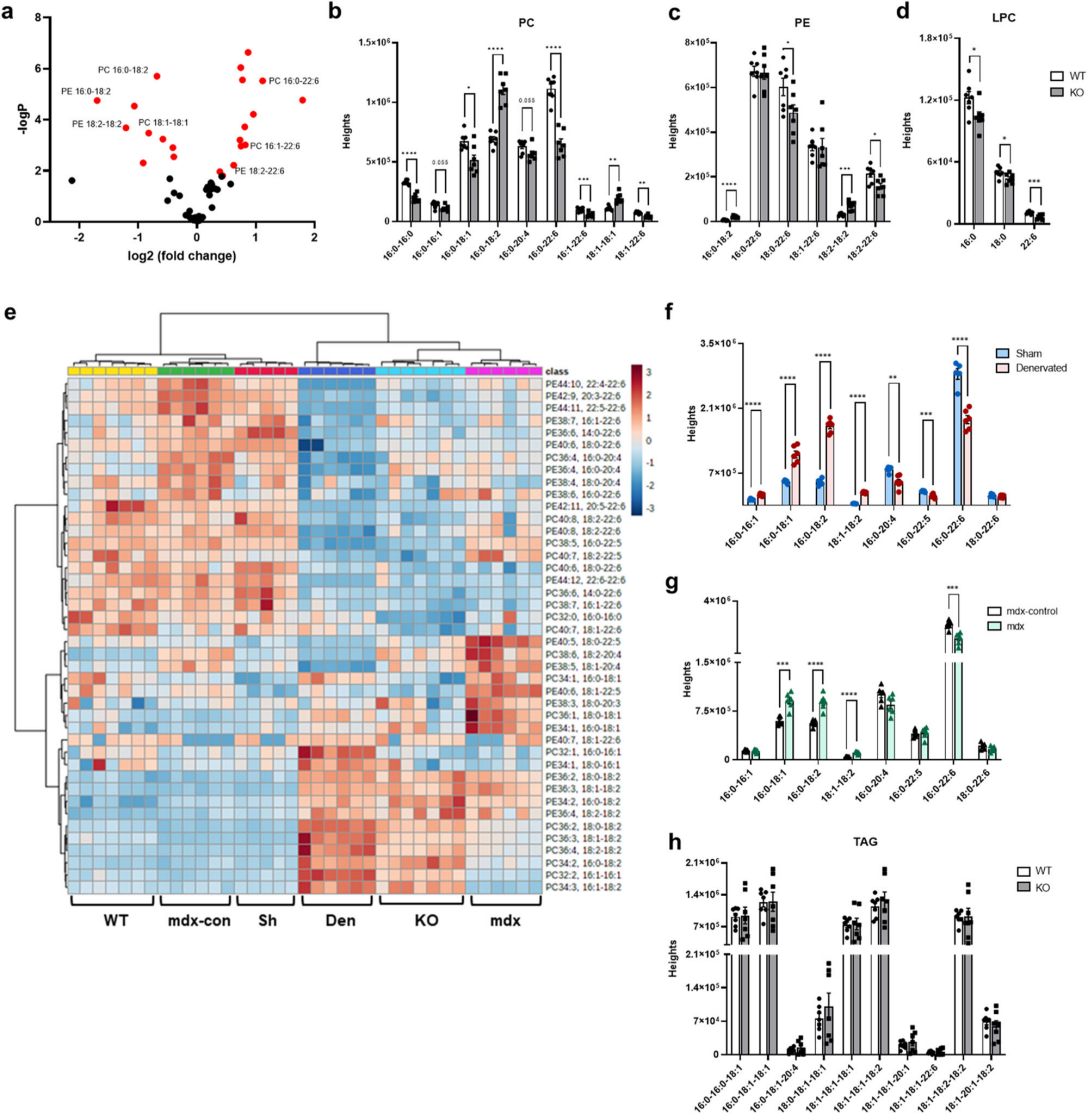
Comparison of phospholipid profile of the skeletal muscle from Gde5 skKO mice with denervated and mdx mice. **a** Volcano plot of phospholipid content in *Gde5* skKO and WT mice skeletal muscle. **b**–**d** Profiles of PC (**b**), PE (**c**), and LPC (**d**) molecular species in *Gde5* skKO and WT mice skeletal muscle (*n* = 7). **e** Heatmap representation of phospholipid species in skeletal muscle of *Gde5* skKO, mdx, denervation and their respective controls. **f**, **g** Profiles of PC molecular species in denervated (**f**) and mdx (**g**) skeletal muscle. **h** TAG molecular species of *Gde5* skKO and WT skeletal muscle under normal chow diet. TAG, triacylglycerol. Values are means ± SEM. Statistical analysis was performed with Student’s *t* test. *p < 0.05; **p < 0.01; ***p < 0.001; ****p < 0.0001.

Phosphatidylcholine (PC) is a zwitterionic phospholipid that constitutes between 30% and 60% of mammalian cell membrane phospholipids. Since GPC is an intermediate in phospholipids metabolism, we next investigated how GDE5 deficiency with GPC accumulation affects PC metabolism by performing lipidomic analysis. Although no difference was observed in the amounts of total PC and PE between *Gde5* skKO and WT mice (Fig. S8a-c), we found increased linoleic acid-rich PC (16:0–18:2) and decreased DHA-rich PC (16:0–22:6) levels in the *Gde5* skKO muscles compared to those in the WT (Fig. 4a, b). These data suggest a mechanistic relationship between GPC accumulation and PC remodeling in skeletal muscle following GDE5 deficiency. A similar pattern was observed in PE (Fig. 4c), and lysophosphatidylcholine (LPC) 16:0, 18:0, and 22:6 were significantly lower in *Gde5* skKO mice (Fig. 4d). Next, to exclude a possibility of the side-effect of HSA-Cre transgene on skeletal muscle functions, we confirmed that the constitutive expression of Cre recombinase in the muscle had no effect on muscle contraction functions, choline metabolite levels and PC levels (Fig. S9). PC biosynthesis shares core intermediates, such as PA and diacylglycerol (DAG), with the triglyceride (TAG) biosynthesis pathway^26^. Although there were no differences in both total TAG levels (Fig. S8d) and the TAG profile within the skeletal muscles of the *Gde5* skKO mice that were fed a normal diet (Fig. 4h), we observed similar PC compositional changes, decreased total TAG level, and altered TAG profiles in response to the HFD treatment, particularly involving the C18:1 fatty acid (Fig. S8e–i).

**Fig. 5:**
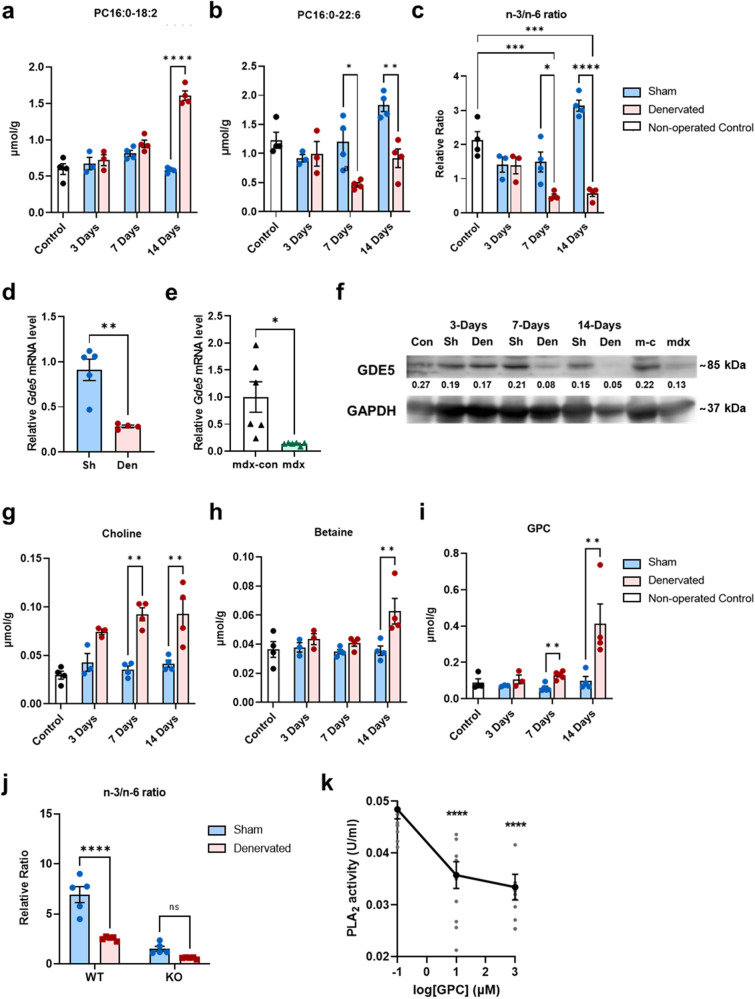
Role of GPC in modulating PC composition of the skeletal muscle. **a**–**c** PC compositional change post-denervation with non-operated control as external control and sham-operated muscle as internal control. **d**, **e** qPCR analysis of *Gde5* mRNA expression in denervated (**d**) and mdx (**e**) skeletal muscle compared to their respective control. Sh, sham; Den, denervated; mdx-con, mdx control. **f** GDE5 western blot on denervated and mdx skeletal muscle compared to their respective control. Con, non-operated control; Sh, sham; Den, denervated; m-c, mdx control. **g**–**i** Choline metabolites levels post-denervation with non-operated control as external control and sham-operated muscle as internal control. **j** n-3/n-6 ratio of denervated *Gde5* skKO and WT skeletal muscle compared to respective sham-operated muscle. **k** PLA_2_ activity in skeletal muscle homogenates following addition of GPC (*n* = 6). Values are means ± SEM. Statistical analysis was performed with Student’s *t* test. *p < 0.05; **p < 0.01; ***p < 0.001; ****p < 0.0001.

Phospholipid compositional alterations have been discussed in muscle atrophy models^27,28^; consequently, we compared the phospholipid composition of *Gde5* skKO mice with two mouse models of atrophy, namely, denervation and mdx mice. Surprisingly, we observed similar patterns in these three models, wherein linoleic acid-rich species increased and DHA-rich species decreased (Fig. 4e-g and S8j). Furthermore, the altered PC composition was aggravated by a longer duration of denervation (Fig. 5a, b), accompanied by a gradual decrease in n-3/n-6 ratio (Fig. 5c), prompting us to consider a similar mechanism for PC composition regulation. We next assessed GDE5 expression at the mRNA (Fig. 5d, e) and protein levels (Fig. 5f, S10) in the atrophy models to clarify whether GDE5 was involved in PC compositional changes. GDE5 expression was significantly decreased in both models compared to their respective controls. In addition, a time-dependent GPC accumulation was observed 7 days post-denervation (Fig. 5i), in parallel with decreases in n-3/n-6 ratio (Fig. 5c) and GDE5 expression (Fig. 5f and S10). These findings suggest that the change in PC composition is mechanistically linked to decreased GDE5 function and possibly GPC accumulation, while it may also be associated with muscle weakness. Although an increase in the n-3/n-6 ratio was observed in the contralateral sham-operated limb 14-days post-denervation in the absence of any increase in GDE5 expression (Fig. 5c), this could be attributed to muscle overuse, as the mice were dependent on the sham-operated limb for movement. Exercise training has been reported to promote increases in DHA-rich PC in skeletal muscle^16,29,30^, and it is plausible that the compensatory use of sham-operated limbs has similar effects. Given that training induces complex metabolic processes, it is feasible that other processes are also involved in regulating the n-3/n-6 ratio of trained muscle without altering GDE5 expression. However, the mechanisms associated with the regulation of PC compositional change in trained muscles require future investigation. In contrast to *Gde5* skKO, elevated choline and betaine levels were observed in the denervation model 7-days and 14-days post-surgery, respectively (Fig. 5g, h).

Previously, the n-3/n-6 balance in the membrane has been shown to correlate with muscle function^30,31^. As denervation led to parallel changes in the n-3/n-6 ratio, GDE5 expression, and GPC levels, we denervated both *Gde5* skKO mice and their WT littermates to verify GDE5 involvement in regulating the n-3/n-6 balance, which revealed a significant decrease in the n-3/n-6 ratio in WT mice alone (Fig. 5j). Given that GDE5 deficiency restored the changes in the n-3/n-6 ratio induced by denervation, we examined the effect of GPC accumulation on PLA_2_, an enzyme involved in the PC remodeling pathway (Lands’ cycle)^32^, to investigate the mechanism through which GDE5 participates in regulating PC composition. We examined PLA_2_ activity in the skeletal muscle in the presence of GPC, revealing complete inhibition of PLA_2_ activity upon the addition of GPC in vitro, even at low doses (Fig. 5k). Collectively, these results emphasize the importance of GDE5 in regulating PC composition, particularly the n-3/n-6 balance, through negative feedback of GPC on PLA_2_ enzyme. This shed more light on the significance of PC composition in muscle function.

### The balance of n-3/n-6 in *Gde5* skKO mice may correlate with alterations in contractile properties

**Fig. 6:**
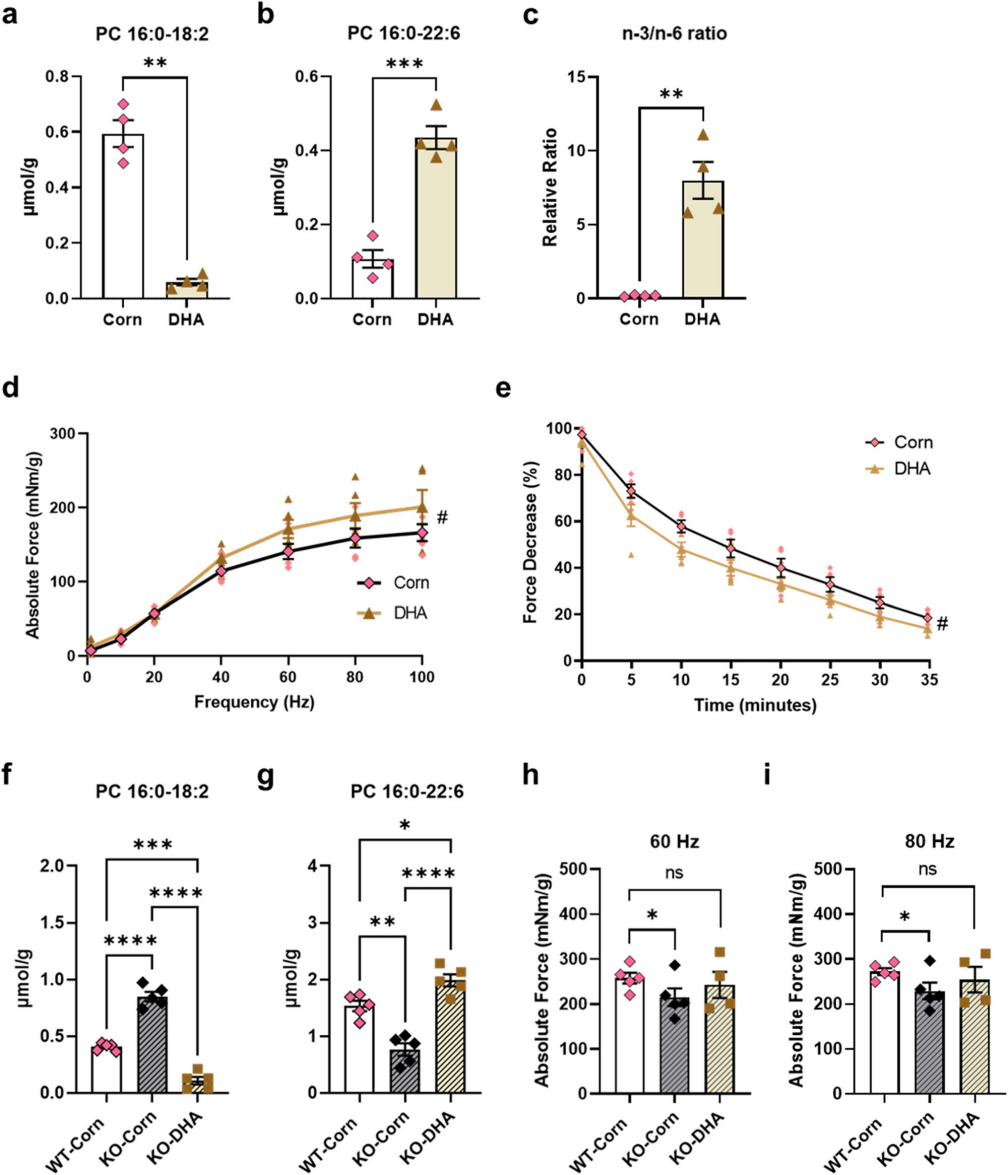
DHA-rich diet affects phospholipid profile of the skeletal muscle and contraction force of mice. **a**–**c** PC compositional change in skeletal muscle of mice fed with DHA-rich and corn oil-rich diet. **d** Contractile force test of mice fed with DHA-rich and corn oil-rich diet (*n* = 5). **e** Fatigability test of mice fed with DHA-rich and corn oil-rich diet (*n* = 5). **f**, **g** PC compositional change in skeletal muscle of *Gde5* skKO fed with DHA-rich and corn oil-rich diet with WT fed with corn-oil rich diet as control. **h**, **i** Contraction force test of *Gde5* skKO fed with DHA-rich and corn-oil rich diet at 60 Hz (**h**) and 80 Hz (**i**). Values are means ± SEM. Statistical analysis was performed with Student’s *t* test (**a**–**c**) and two-way ANOVA (**d**–**i**), followed by Tukey’s test (**f**–**i**). #*p* < 0.05 (main effect); *p < 0.05; **p < 0.01; ***p < 0.001; ****p < 0.0001.

On the basis of the similarities in skeletal muscle lipid profiles between *Gde5* skKO mice and atrophy mouse models, we investigated whether contractile force and fatigability are affected by PC compositional changes. We fed WT mice with a DHA-rich diet for 2 weeks to induce a PC compositional change to n-3 rich species and an increase in n-3/n-6 ratio in the skeletal muscle; a corn-rich diet was fed to the control group. We observed an increase in DHA-containing PC and an elevated n-3/n-6 ratio (Fig. 6a–c). Consistent with the results from the *Gde5* skKO mice, the high DHA content and n-3/n-6 ratio in PC yielded opposing results with respect to contractile force and fatigability (Fig. 6d, e). We further fed the *Gde5* skKO mice with a DHA-rich diet for 2 weeks and observed an increase in contractile force within the muscles, accompanied by an increase in the level of DHA-containing PC and an elevated n-3/n-6 ratio (Fig. 6f–i). These observations underscore the involvement of GDE5 in modulating PC composition, especially the n-3/n-6 balance, suggesting that PC composition is correlated with muscle weakness.

### PC compositional change increases the open probability of RyR and potential Ca^2+^ leakage from the sarcoplasmic reticulum

**Fig. 7:**
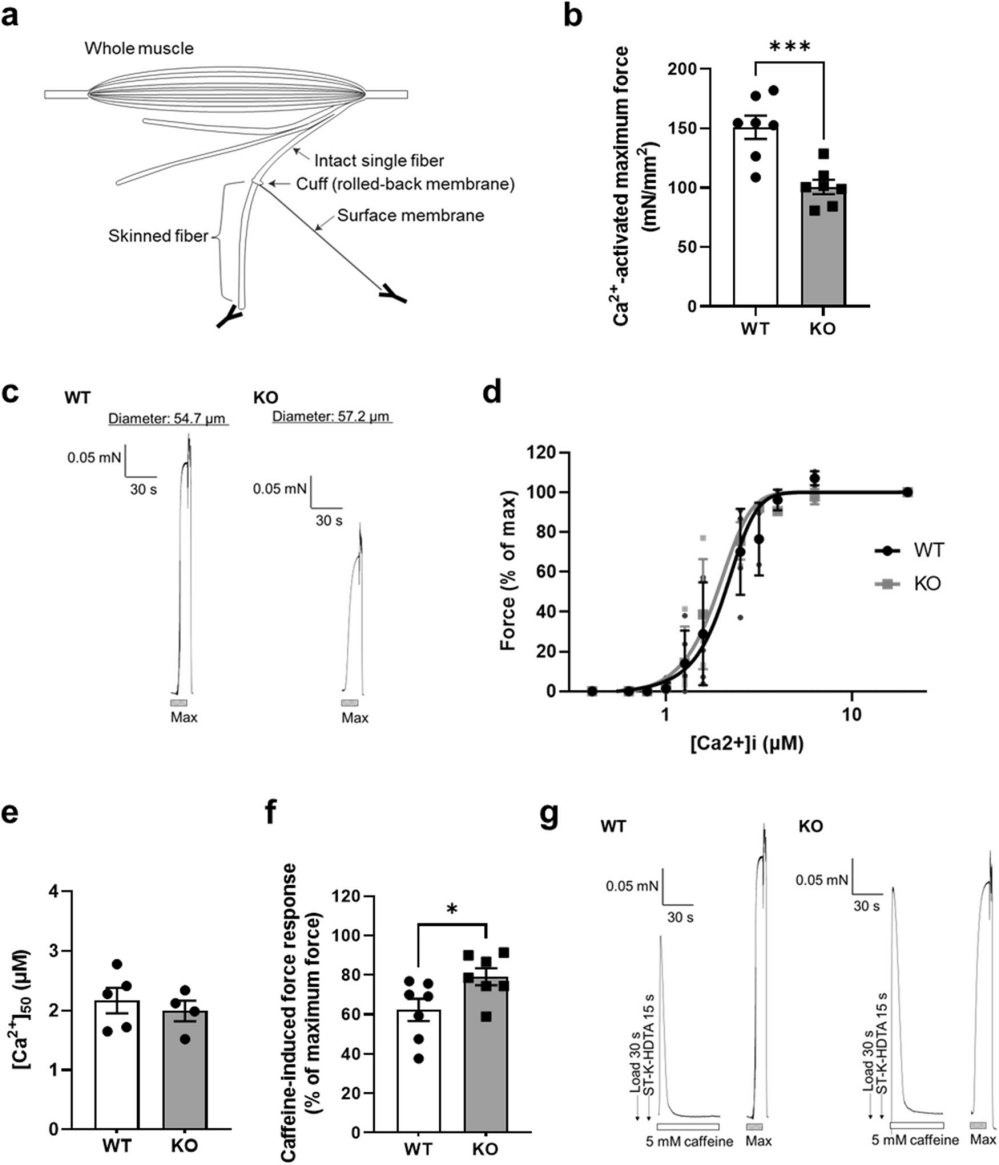
Skinned fiber experiments with the skeletal muscle from *Gde5* skKO and WT mice. **a** Scheme of skinned fiber preparation. **b** Ca^2+^-activated maximum force of *Gde5* skKO and WT skinned fiber. **c** Representative example of Ca^2+^-activated maximal force in skinned fiber. **d** Force-Ca^2+^ curve of *Gde5* skKO and WT skinned fiber (*n* = 4–5). **e** [Ca^2+^]_50_, Ca^2+^ concentration required to 50% of maximal force in skinned fiber of *Gde5* skKO and WT mice. **f** Caffeine-induced force response in skinned fiber of *Gde5* skKO and WT mice. **g** Representative example of caffeine-induced force in skinned fiber. Values are means ± SEM. Statistical analysis was performed with Student’s *t* test. *p < 0.05; ***p < 0.001.

To identify the molecular mechanisms underlying the decreased contractile force of skeletal muscle in *Gde5* skKO mice, we isolated single muscle fibers from the gastrocnemius (GAS) using fine forceps, and a segment of the skinned fiber was used to analyze the myofibril and sarcoplasmic reticulum (SR) properties (Figs. 7a and S11a). The DHA content of the SR membrane is associated with SR Ca^2+^ ATPase (SERCA) function and SR Ca^2+^ permeability^33^, and previous studies have showed that SERCA activity decreases during muscular dystrophy^34^. Based on this information, we first evaluated SR Ca^2+^ uptake ability by exposing the skinned fiber to a load-release cycle, but did not observe any difference (Fig. S11b). This may be attributed to the SERCA activity and ionophore ratio, an indicator of SR Ca^2+^ permeability, which did not differ compared to WT (Fig. S11c, d), implying that the muscle weakness in *Gde5* skKO mice was not due to changes in SERCA function or SR Ca^2+^ permeability. Next, we examined the myofibril cross-bridge function by measuring Ca^2+^-activated maximum force, which was significantly reduced in *Gde5* skKO muscles compared to WT muscles (Fig. 7b, c). The cross-bridge function is associated with force production^35^; therefore, this data is consistent with our in vivo experiment. We also evaluated myofibril Ca^2+^ sensitivity, which typically decreases in fatigued muscle^36^, but our results showed no differences between the groups (Fig. 7d, e), indicating that Ca^2+^ sensitivity in the myofibrils was unaffected by *Gde5* skKO. In addition, a lower contractile force is linked with decreased Ca^2+^ release from the SR^37^; thus, we next assessed the function of ryanodine receptor (RyR)—the main channel for SR Ca^2+^ release—by measuring the caffeine-induced force response. Caffeine is a potent Ca^2+^ releasing agent specific for RyR and has been used as an indicator of open probability of RyR^38^. *Gde5* skKO induced a higher open probability of RyR (Fig. 7f, g), indicating greater Ca^2+^ leakage via RyR, which is consistent with previous reports linking higher RyR open probability and elevated Ca^2+^ leakage from the SR^39^. Increased Ca^2+^ leakage may have contributed to the lower force in *Gde5* skKO mice because it can decrease SR Ca^2+^ content and increase cytoplasmic Ca^2+^ concentration. Thus, we deduce that the in vivo low contractile force in *Gde5* skKO mice is partly caused by impaired myofibril cross-bridge function, and that increased open probability of the RyR may contribute to force depression.

## Discussion

GPC is widely recognized as a major form of intracellularly stored choline and an organic osmolyte. In mammals, GPC levels may be tightly controlled by phospholipase activity via PC degradation pathway as no transporter for GPC has been reported. However, the in vivo molecular mechanism of GPC homeostasis remains largely unclear. We previously reported that GDE5 selectively hydrolyzes GPC into choline in vitro*;* in the present study, we confirmed that GDE5 deficiency led to GPC accumulation in various tissues, strongly suggesting its role in the PC degradation pathway. Membrane phospholipid composition has been well-documented to affect membrane fluidity and signal transduction associated with physiological conditions^12–14^. In particular, PC metabolism and composition have attracted significant attention; however, it is largely unclear how PC compositional changes impact physiology because it is difficult to introduce specific changes in PC composition in mammals. In skeletal muscles, PC composition changes following physical exercise and diet composition, and may be correlated with muscle function^15–17^. Here, we provide evidence that inactivation of the GDE5 enzyme induces PC compositional changes in skeletal muscle, suggesting that the intracellular GPC level plays a key role in regulating PC composition.

The diversity of PC fatty acid composition is established through the remodeling pathway (Lands’ cycle) and the Kennedy pathways in mammals^40^. PC homeostasis is closely modulated by remodeling reactions known as the Lands’ cycle. In this process, PC is cleaved by PLA_2_ at the sn-2 position to produce LPC, which is then re-acylated to form new or different PC species by lysophospholipid acyltransferases (LPLATs)^32,41^. Furthermore, recent studies using mouse genetic models have shown that LPLATs, which incorporate fatty acyl chains into the sn-2 position of PC, play important roles in the PC fatty acid composition^42,43^. GPC reportedly acts as a competitive inhibitor of lysophospholipase activity in brain homogenates in vitro^44^. In this study, we found that a high GPC concentration suppressed PLA_2_ activity, and the lipidomic profiles in *Gde5* skKO skeletal muscle showed significantly reduced levels of LPC 16:0, 18:0, and 22:6. Consequently, this proposes a novel role for intracellular GPC, suggesting its involvement not only in maintaining a choline source for PC biosynthesis, but rather in modulating PC composition through the regulation of key enzymatic activities in the PC remodeling pathways, including the Lands’ cycle. Additionally, in yeast, phospholipase B-mediated hydrolysis of PC results in GPC formation, which can be re-acylated to PC by GPC acyltransferase (Gpc1) and LPC acyltransferase. Interestingly, Gpc1 activity regulates the PC species balance between monounsaturated and diunsaturated PC species^45,46^. However, PC fatty acid composition is also regulated by the Kennedy pathway. Gro3P, the precursor of this pathway, is converted to lysophosphatidic acid (LPA) by Gro3P acyltransferases (GPATs), which is subsequently transformed into PA—a common intermediate for PC and TAG—by LPA acyltransferases (LPAATs)^47^. An intriguing finding is that the muscle TAG level in *Gde5* skKO mice was decreased following HFD intake. Together with the striking accumulation of Gro3P, these results suggest a Kennedy pathway downregulation under GDE5 deficiency. Specifically, LPAAT3 is reportedly selective in incorporating C22:6 into LPA, which eventually determines the levels of DHA-rich phospholipids^48^. Therefore, we cannot exclude the possibility that PC compositional changes are also mediated by Kennedy pathway downregulation. Notably, denervated muscles exhibited an increase in muscle choline levels 7 days post-surgery, although tissue choline levels are supposedly constant under normal conditions. These data suggest that the PC synthesis pathway is downregulated in denervated muscles and that the denervation-induced PC compositional changes are caused by both impaired PC degradation and the PC synthesis pathways. Further investigations are necessary to elucidate the role of intracellular GPC in modulating the two PC remodeling pathways, namely, the Lands’ cycle and Kennedy pathways.

The functional relationship between membrane phospholipid composition and skeletal muscles has been discussed extensively^16,49,50^. For instance, DHA supplementation can alter the membrane phospholipid composition in skeletal muscle and improve fatigue resistance^51–53^. However, because diet composition can affect systemic metabolic changes in the body, it is unclear whether the effect on muscle was exclusively related to changes in PC composition or whether it was due to secondary effects of the altered overall metabolism. In this respect, our study provides interesting information on how PC composition may be related to muscle function. *Gde5* skKO mice showed decreased PUFA levels and the n-3/n-6 ratio in the phospholipid membrane, which was also observed in denervated and mdx muscles. Furthermore, increasing the DHA-containing PC level and n-3/n-6 ratio using a DHA-rich diet in WT mice enhanced contractile force. This finding signifies the relationship between PC fatty acid composition, especially the n-3/n-6 balance, and muscle strength, and provides mechanistic insights into muscle weakness in denervated and mdx mice. Additionally, the *Gde5* skKO mice exhibited a higher resistance to muscle fatigue. While this result appears contradictory, it aligns with the slower glycogen degradation rate in *Gde5* skKO skeletal muscles. Notably, several studies have demonstrated a strong correlation between glycogen levels and muscle fatigue^54–56^. A previous study in humans demonstrated that slower glycogen utilization improves endurance capacity^57^. As a major energy source in skeletal muscles, glycogen is possibly linked to muscle fatigue through its role in providing ATP, which is necessary for muscle contractions^58^. Furthermore, under low glycogen levels, muscle fibers possibly lose their ability to respond to transverse tubular depolarization, even in the presence of ATP^59^. Hence, the slower glycogen utilization observed in *Gde5* skKO mice may play metabolic and structural roles in the context of muscle fatigue.

Low force production in skeletal muscle is reportedly associated with decreased SR Ca^2+^ release^37,60^. Our skinned fiber experiment showed a larger SR Ca^2+^ leakage through the RyR in *Gde5* skKO muscles without changes in the activity of the SERCA pump. This may have contributed to reduced Ca^2+^ release from the SR, according to previous observations, where a leaky RyR resulted in decreased SR Ca^2+^ release^39,61,62^. Furthermore, elevated intracellular Ca^2+^ can activate calpain, member of a family of calcium-activated proteases, that mediates cytoskeletal and myofibril proteolysis, leading to reduced force production post-exercise^63–65^. The RyR-mediated Ca^2+^ leakage in *Gde5* skKO may have induced calpain activation, leading to impaired cross-bridge function and reduced force production. Leakage of Ca^2+^ from the RyR also reportedly occurs in denervated and mdx muscles^61,66^, which show PC compositional properties similar to the muscles of *Gde5* skKO mice used in this study. Previous reports showed a reduced interaction between RyR and FKBP12, a calcium channel stabilizing binding protein, which contributed to altered Ca^2+^ homeostasis in mdx muscles^61^; thus, a functional relationship may exist between altered PC composition and alterations in the macromolecular complex containing RyR in the *Gde5* skKO muscles. Although decreased neuromotor activity and reduced acetylcholine levels are the primary factors contributing to muscle alterations following denervation^67^, our study suggests that decreased levels of n-3 PUFAs and a lower n-3/n-6 ratio in the phospholipid membrane partly play a pathological role in denervated muscle.

Another intriguing finding of this study was the altered glucose metabolism following GDE5 downregulation, indicating a possible interaction between choline/GPC and glucose metabolism. In this study, insulin sensitivity was downregulated following GDE5 suppression. Both in vitro and clinical studies showed that an increase in PUFAs in membrane PC was associated with improved insulin sensitivity^68,69^, suggesting that membrane PC composition modulates insulin receptor activity at the cell surface. Interestingly, despite the lowered insulin sensitivity in *Gde5* skKO muscles, several glycolysis-related metabolites, including G6P, DHAP, and Gro3P, were significantly increased, along with the glycogen level. Previous studies on skeletal muscle-specific GLUT4 KO mice have showed similar characteristics to *Gde5* skKO mice, where insulin resistance, glycogen accumulation, increased hexokinase activity, and elevated G6P levels were observed. Although GLUT4 deficiency in skeletal muscle reduces glucose transport, it elevates glycogen content through two other mechanisms. First, glycogen-targeting subunits of protein phosphatase-1 activate glycogen synthase. Second, glycogen synthase is allosterically activated by an increase in G6P due to higher hexokinase activity^70,71^. As membrane PC composition can affect membrane fluidity, the PC compositional changes in *Gde5* skKO mice may affect GLUT4 translocation or its activity on the plasma membrane, leading to altered glucose transport and metabolism, as seen in GLUT4 KO mice. Alternatively, GDE5 may directly regulate glycogen metabolism. GDE5 is a cytosolic protein with an N-terminal carbohydrate-binding domain often found in glycosyl hydrolases and intracellular glycogen-binding proteins^7^. Glycogen degradation leads to elevated glucose-based glycogen phosphate due to the faster removal of glucose units compared to phosphate^21^. *Gde5* skKO muscles showed reduced glycogen C6-phosphate levels, indicating that elevated glycogen levels in the *Gde5* skKO muscles might be a consequence of a decreased glycogen degradation rate. Consecutively, this higher glycogen content may result in phenotypes similar to those seen under GLUT4 deficiency. Although the underlying mechanism of increased glycogen levels despite reduced insulin sensitivity in *Gde5* skKO muscles remains unclear, these observations prompt us to consider that the GDE5 protein, with its N-terminal carbohydrate-binding domain in addition to GPC hydrolyzing activity, has alternative enzymatic activity in glycogen metabolism.

**Fig. 8:**
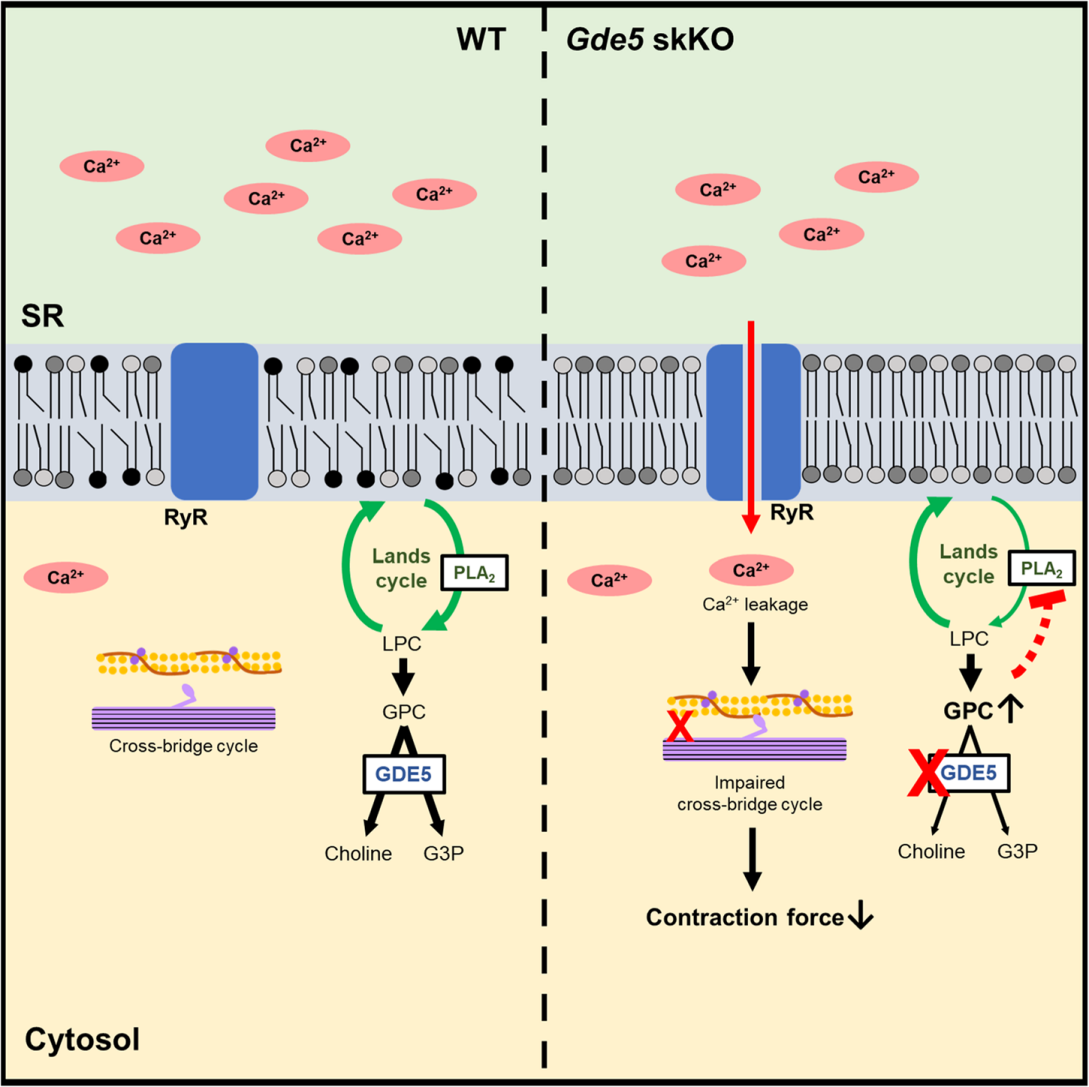
A schematic presentation of reduced passive force in skeletal muscles of GDE5/Gpcpd1-deficient mice. The loss of GDE5 resulted in a significant accumulation of GPC and reduced levels of glycerophospholipids that bind DHA via negative feedback on PLA_2_ activity. The alterations in glycerophospholipids were accompanied by an increase in the open probability of ryanodine receptor and lower maximum Ca^2+^-activated force, indicative of impaired cross-bridge cycle, which contribute to the decreased contractile force in the muscles.

To summarize, our study highlights the physiological role of GPC metabolism in the regulation of PC composition and how it is associated with skeletal muscle properties and glucose metabolism (Fig. 8). Because GPC is mainly generated from PC degradation, it is plausible that it is not merely an intermediate, but a key element involved in the modulation of phospholipid membrane metabolism and turnover. Phospholipid membrane properties are crucial for cell functions, which is probably why GPC accumulation leads to various metabolic and functional changes within the muscles. These findings could have broader implications for understanding a variety of disorders related to aberrant choline metabolism and disrupted phospholipid membrane metabolism, not only in skeletal muscle-related pathologies, but also those affecting other tissues.

## Methods

### Mouse models

All animal experiments were conducted in accordance with the animal care protocol approved by the Animal Use Committee of the Hiroshima University (Ethical approval No. C22-30) and we have complied with all relevant ethical regulations for animal use. We previously created whole-body *Gde5* KO mice harboring exon 11-deleted alleles by using CRISPR-Cas9 ribonucleoproteins to target the both regions outside the exon 11 in *Gde5*^72^. The *Gde5*^+/−^ F1 male offspring from this breeding were then backcrossed with purebred C57BL/6 J females (Charles River, Japan) to obtain F2 offspring; this process was continued until the F3 mice were obtained. Then, 8-week-old male *Gde5*^+/−^ mice were fed with control diet (AIN-93G diet) or choline-deficient diet (AIN-93G diet with no choline) for 8 weeks. For generation of *Gde5*-floxed mice, we targeted exon 11 of *Gde5*. Floxed mice were created using CRISPR–Cas9 and PITCh (Precise Integration into Target Chromosome) system with microhomology-mediated end joining-directed plasmid as a donor. The C57BL/6 zygotes were cultured for 3 h. We performed microinjection of CRIS-PITCh (v2) donor vector and Cas9 ribonucleoprotein containing two gene-specific gRNAs (Left: 5′-GGAATGACTGACACACCAAC-3′, Right: 5′-GGTGTCAGATCCTGTACACT-3′) and one generic gRNA (5′-GTGCTTCGATATCGATCGTT-3′) targeting the donor vector by co-delivery of in vitro transcribed Trex2 mRNA to inhibit large chromosomal deletion. After microinjection, the zygotes were transferred to pseudopregnant female ICR mice. We selected knock-in mutants and investigated whether the vector DNA was accidentally integrated into the genome. PCR amplification of the backbone sequence was performed to detect genomic integrants. *Gde5*^flox/flox^ mice were crossed with skeletal muscle-specific Cre recombinase (human α-skeletal actin (HSA)-Cre) mice to generate skeletal muscle-specific GDE5-deficient (HSA-Cre;*Gde5*^flox/flox^) mice. Male HSA-Cre;*Gde5*^flox/flox^ mice with ages of 15- to 18-week-old were used in contractile force and fatiguability test with *Gde5*^flox/flox^ littermates as controls. In glucose tolerance test (GTT) and fasting challenge, male offspring and its *Gde5*^flox/flox^ littermates with ages of 12- to 15-week-old were used. For denervation model, male C57BL/6J mice aged 8-week-old (~24 g) were obtained from Charles River. All animals were housed at 24-26 °C in a 12 h light-dark cycle (8:00-20.00 light cycle, 20.00-8.00 dark cycle) and fed with commercial chow diet (MF, Oriental Yeast Co., Ltd.) and water ad libitum.

### RNA extraction and qPCR analysis

Total RNA was extracted using QIAzol and purified with a RNeasy Lipid Tissue Mini Kit (Qiagen Sciences, Germantown, MD, USA). Reverse transcription was conducted with ReverTra Ace^TM^ (TOYOBO, Osaka, Japan), random primers (TOYOBO), and dNTPs (TOYOBO). For qPCR analysis, cDNA and primers were added to the THUNDERBIRD SYBR qPCR Mix (TOYOBO). PCR reactions were then performed using StepOnePlus^TM^ (Applied Biosystems, Foster City, CA). The primers used can be found in the Supplementary Table 1.

### Insulin tolerance test

*Gde5* skKO mice were fasted 6 h prior to the experiments. Insulin (Eli Lilly, Japan) at a dosage of 0.5 U/kg was intraperitoneally administered and blood glucose was measured using Accu-Chek (Roche Diabetes Care) at 0, 15, 30, 60, 90, and 120 min after the injection.

### Muscle glycogen

Excised gastrocnemius (GAS) muscles were separated into deep and superficial region visually by color. Approximately 10 mg of superficial region was obtained and lysed in 2 N HCl at 100 °C for 2 h and then neutralized with 2 N NaOH. Samples were centrifuged and supernatants were used for measurement. The assay mixture was composed of 50 mM Tris-HCl (pH 8.1), 1 mM MgCl_2_, 0.5 mM ATP, 0.5 mM NADP, 0.14 U/ml glucose-6-phosphate dehydrogenase, and 0.28 U/ml hexokinase. Absorbances were measured on 340 nm wavelength and glycogen contents were calculated based on the obtained standard curve. Each sample was replicated 3 times and the average of three measures was used for data analysis.

### Muscle glycogen C6 phosphate

Glycogen C6 phosphate was quantified as an established proxy for total phosphate esterification of glycogen. First, glycogen was extracted essentially as previously described^73^. Briefly, frozen GAS muscles were ground in liquid nitrogen and boiled in 30% [w/v] KOH and precipitated once in 67% ethanol with 15 mM sodium sulfate (18 h at -20°C). Redissolved glycogen was subsequently precipitated thrice in 67% [v/v] ethanol and 15 mM LiCl (at least 1 h at -20°C). Aliquots of the redissolved glycogen were subjected to (1) glycogen quantification via enzymatic glucose quantification following incubation with amyloglucosidase (Megazyme) and (2) to acid hydrolysis. Glycogen C6 phosphate was determined by measuring glucose-6-phosphate (G6P) in neutralized hydrolysates of glycogen samples using the enzymatic cycling assay previously described^74^.

### Hexokinase activity

Ten percent GAS muscle homogenates were prepared with extract solution composed of 175 mM KCl, 10 mM GSH, and 2 mM EDTA. Homogenates were freeze-thawed 3 times using liquid nitrogen and centrifuged at 12,500 rpm for 10 min. Supernatants were used for measurement. The assay mixture contained 50 mM triethanolamine, 5 mM EDTA, 8 mM MgCl_2_, 1.3 mM NADP, 2 mM ATP, 2 mM glucose, and 7 U/ml of glucose-6-phosphate dehydrogenase. Absorbances were measured at 340 nm every 30 s for 240 s and hexokinase activity was calculated based on the obtained standard curve. Each sample was replicated 3 times and the average of three measures was used for data analysis.

### DNA microarray

Total RNA from the GAS muscle of WT and *Gde5* skKO mice at 15 weeks of age was isolated using RNeasy Lipid Tissue Kit and subjected to cRNA synthesis. Fluorescence labeling, hybridization, and image processing were performed according to the manufacturer’s instructions (Agilent Technologies, Palo Alto, CA). Briefly, cRNAs were fragmented and hybridized on the 44 K whole mouse genome oligo microarray slides at 65 °C for 17 h. The glass slides were then washed and scanned using Agilent DNA microarray scanner (Agilent Technologies) as previously described^7^. Gene expression data were obtained by the Agilent Feature Extraction Program (version 9.5). DNA microarray data are deposited in the NCBI GEO data base (www.ncbi.nlm.nih.gov/geo) under accession number GSE262199.

### Denervation

To induce atrophy to the GAS muscles, 8-week-old male C57BL/6 mice were subjected to hindlimb denervation. Sixteen mice were divided into 4 groups of control, 3-days, 7-days, and 14-days post-surgery group. Mice were anesthetized using 1.5-2% inhaled isoflurane during surgery. Approximately 5 mm section of the sciatic nerve in the right hindlimb of mice were cut, and the left hindlimb was sham-operated. Non-operated mice were used as the external control to avoid potential physiological effect of denervation on its contralateral limb. Denervated mice were dissected after the respective post-surgery period. GAS muscles of both limbs were obtained, weighed, and stored for biochemical analyses.

### Total cellular membrane protein extraction

Total cellular membrane proteins were extracted according to ab65400 Plasma Membrane Protein Extraction Kit (Abcam). Briefly, skeletal muscles were minced and homogenized in homogenize buffer mix containing buffer and protease inhibitor cocktail (1/500). Homogenates were aliquoted into 1.5 mL tubes and centrifuged in 700 ×*g* at 4 °C for 10 min. Supernatants were obtained and further centrifuged in 10,000×*g* at 4 °C for 30 min. Pellets were obtained as total cellular membrane proteins and resuspended in RIPA buffer for further analysis.

### Western blot

Ten percent GAS muscle homogenates were prepared with RIPA buffer containing 1 mM PMSF, 20 µg/ml aprotinin, and 10 µg/ml phosphatase inhibitor cocktail. Homogenates were centrifuged at 13,000 g for 15 min and the supernatants were used for measurement. Total cellular membrane protein extracts described previously were used for GLUT4 measurement. Protein concentration was quantified using BioRad *DC* Protein Assay (Bio-Rad Laboratories, Inc). 15 µg protein for each sample was prepared in sample buffer composed of SDS, glycerol, and methanol, then boiled in 92 °C for 3 min. Prepared samples were then loaded into the wells of the SDS-PAGE gel and run for 100 min at 24-26 mA. Thereafter, protein was transferred to PVDF membranes using semi-dry method at 2 mA x membrane area for 120 min. The membranes were next blocked using 4% skim milk in 1x PBS for 1 h, then incubated in primary antibody overnight at 4 °C for anti-GDE5 (1:1000, Okazaki et al. 2010), anti-GAPDH (1:5000, WAKO, 016-25523), anti-GLUT4 (1:2000, Proteintech, 66846-1-lg), and anti-caveolin-1 (1:1000, Transduction Laboratories, c13630). Next, membranes were washed 3 times in PBS-T, then incubated with respective secondary antibodies. Can Get Signal™ Immunoreaction Enhancer Solution (TOYOBO, NKB-201 and NKB 301) were used to incubate antibodies. Protein bands were detected using Western Lighting-ECL (PerkinElmer, Inc).

### PLA_2_ activity assay

GAS muscle was homogenized in buffer (1:9) containing 1% Triton-X, 50 mM Tris-HCl (pH 8.0), 150 mM NaCl, protease inhibitor cocktail (1:200) and phosphatase inhibitor cocktail (1:200). Homogenates were centrifuge at 13,000×*g* for 15 min and supernatants were used for assay using EnzCheck® Phospholipase A_2_ assay kit (Invitrogen).

### Contractile force and fatigability test

Contractile force and fatigability tests were conducted on electrically stimulated intact gastrocnemius (GAS) muscles. Mice were anesthetized by intraperitoneal administration using a mix of Domitor® (Orion Pharma Animal Health), Midazolam (Sandoz), and Vetorphale® (Meiji Seika Pharma) at 10 µL/mg body weight. Electrodes were attached to the left GAS muscle using tapes. Then, the animal was placed in a supine position and the left limb was attached to a homemade foot holder connected to an isometric transducer. The GAS muscles were stimulated at 100 Hz, 80 Hz, 60 Hz, 40 Hz, 20 Hz, 10 Hz, and 1 Hz for 1.5 s each with 1 min interval from each frequency. Five minutes after the last frequency, the GAS muscles were stimulated at 70 Hz for 0.35 s repeated at an interval of 9 s for the first 5 min and the interval was reduced every 5 min to 8, 7, 6, 5, 4, and 3 s. Limb movement during stimulation was recorded on a personal computer and analyzed using Lab-Chart software (version 7, ADInstruments, Japan). Both stimulated and unstimulated muscles were immediately excised afterwards for skinned fiber experiment and biochemical analyses.

### Skinned fiber experiment

The superficial region of GAS muscle was pinned out at resting length under paraffin oil and kept on ice. Single muscle fibers were isolated and mechanically skinned by rolling back the sarcolemma with forceps. A segment of the skinned fiber was connected to a force transducer (Muscle tester, SI, Germany), stretched, and transferred to the K-HDTA solution. Skinned fibers were then subjected to measurement of Ca^2+^-activated maximum force, caffeine threshold, and myofibrillar Ca^2+^ sensitivity. All skinned fiber experiments were carried out at 24–26 °C.

### SERCA activity and ionophore ratio

Approximately 30 mg of excised GAS muscles were homogenized on ice in a solution containing 300 mM sucrose, 20 mM Tris-Malate, 0.2 mM phenylmethanesulfonyl fluoride (PMSF), 1.4 µM pepstatin, 2.2 µM leupeptin, and 0.83 mM benzamidine. The homogenates were diluted 4 times in an incubation medium composed of 20 mM HEPES, 200 mM KCl, 15 mM MgCl_2_, 10 mM NaN_3_, and 1 mM EGTA-3 mM Tris. Diluted homogenates were used for measurement. The assay mixture contained 10 mM phosphor(enol)pyruvate, 1 µg/mL calcium ionophore, 0.4 mM NADH, 1 mM CaCl_2_, 20.2 U/mL lactate dehydrogenase (LDH), 12.1 U/mL pyruvate kinase, and 4 mM ATP. Changes in fluorescence were quantified using fluorometric techniques (excitation wavelength, 349 nm; emission wavelength, 410 and 500 nm). Each sample was replicated 3 times and the average of three measures was used for data analysis. Ionophore ratio was obtained by dividing sample measurements in absence of calcium ionophore with measurement in presence of calcium ionophore.

### LC-MS

#### Sample Preparation

The extraction method of Bligh and Dyer was used. GAS muscles were homogenized in H_2_O and added with 2 mL of 2:1 methanol-CHCl_3_ mix, vortexed for 15 s, and stored at −20 °C overnight. On the next day, homogenates were vortexed, then centrifuged at 2000 rpm for 10 min. The remaining pellets were added with 2.5 mL of 2:1:0.8 methanol-CHCl_3_-H_2_O mix, vortexed, and centrifuged at 2000 rpm for 10 min. The supernatants were mixed with previously obtained supernatants and added with CHCl_3_ and H_2_O, 1.3 mL each. The supernatants mixtures were vortexed, and centrifuged at 2000 rpm for 10 min to separate the water-soluble and fat-soluble parts. The water-soluble parts were evaporated at 60 °C for 6 h. The remaining pellets were added with 250 µL H_2_O, vortexed, centrifuged at 2000 rpm for 5 min. 200 µL supernatants were added with 800 µL methanol and stored for measurement. For the fat-soluble parts, evaporation at 40 °C for 3 h were conducted. The remaining pellets were added with the 2:1 methanol-chloroform mix, vortexed, and stored for measurement.

#### PC measurement

Mobile phase A composed of 40% CH_3_CN, 60% dH_2_O, 5 mM HCOONH_4_ (pH 5.0) and mobile phase B composed of 10% CH_3_CN, 90% isopropanol, and 5 mM HCOONH_4_ (pH 5.0) were prepared for measurement. Fat-soluble samples were diluted 50 times in methanol and separated based on gradient generated from mobile phase A and B using Acquity UPLC BEH C18 column (Waters). Ion quantification was performed using ESI method (positive mode) with PC (16:0/18:2) at 60 V cone voltage, m/z = 758.7[MH^+^], 5 V collision voltage, and daughter m/z = 184.2[MH^+^]. PC (17:0/17:0) was used as an internal standard for quantification. The results were adjusted to analyzed muscle weights.

#### Choline, betaine, and GPC measurement

Mobile phase A composed of 95% CH_3_CN, 5% H_2_O, 5 mM HCOONH_4_ (pH 3.0) and mobile phase B composed of 10% CH_3_CN, 90% dH_2_0, 5 mM HCOONH_4_ (pH 3.0) were prepared for measurement. Water-soluble samples were diluted 15 times in solution A and separated based on gradient generated from mobile phase A and B using Acquity UPLC BEH HILIC column (Waters). Ion quantification was performed using ESI method (positive mode) with choline at 25 V cone voltage, m/z = 104[MH^+^], betaine at 25 V cone voltage, m/z = 118[MH^+^], and GPC at 20 V cone voltage, m/z = 258[MH^+^]. The results were adjusted to analyzed muscle weights. Peak area ratios of the analyte were calculated as a function of the concentration ratios of the analyte (QuanLynx, Waters).

### Serum biochemical parameters

Serum alanine transaminase (ALT), aspartate transaminase (AST), cholesterol (T-CHOL), LDL- cholesterol (LDL-C), serum urea nitrogen (BUN), and ketone body and triglyceride (TAG) levels were measured using AU480 analyzer (Beckman Coulter, Krefeld, Germany), which is an instrument for turbidimetric, spectrophotometric and ion-selective electrode measurements.

### Metabolome analysis

GAS muscles from mice were collected on ice, weighed, and transferred into a glass tube. After 2 ml of MeOH, 1 mL of CHCl_3_, 0.7 mL of H_2_O and 5 μL of 1 mg/mL 2-isopropylmalic acid (2-IPM) as an internal standard were added, the mixture was vortexed for 20 min. Then, after 1 mL of CHCl_3_ and 2 mL of H_2_O were added, the mixture was vortexed for 20 min and centrifuged at 500 g for 10 min at room temperature. The upper layer containing water-soluble metabolites was lyophilized using a centrifugal concentrator. The lyophilized sample was sonicated in 40 μL of pyridine containing 20 mg/ml methoxyamine hydrochloride and shook at 1400 rpm for 90 min at 30 °C. Then, 20 μL of *N*-methyl-*N*-trimethylsilyl-trifluoroacetamide was added for derivatization. The mixture was then incubated at 37 °C for 30 min with shaking at 1400 rpm and centrifuged at 21,000×*g* for 5 min at room temperature. In all, 1 μL of the supernatant was injected into a DB-5 capillary column (Agilent Technologies). GC/MS analysis was performed using GCMS-TQ8030 (Shimadzu) equipped with AOC-20i autosampler (Shimadzu). Small molecular weight metabolites was analyzed based on Smart Metabolites Database Release 3.01 (Shimadzu) that contains the data acquisition parameters for 467 compounds in multiple reaction monitoring (MRM) mode. GCMS solution software Version 4.41 (Shimadzu) was used for data processing. Retention time correction was performed based on a standard n-alkane mixture (Restek). Each peak area was normalized by the weight of muscle tissues and the peak area of 2-IPM.

### Lipidome analysis

Lipids were extracted from the muscle tissues that were used in metabolome analysis. The resultant lower layer containing lipids during the extraction process was transferred to a new glass tube and was dried under nitrogen gas stream. Each lipid solution was prepared by adding a solvent mixture (CHCl_3_: methanol = 2:1) which volume was set to be 1 mL/100 mg tissue. Then, 10 μL of the lipid solution was transferred to a new glass tube and evaporated to dryness. The dried sample was dissolved in 100 μL of methanol:toluene containing the following internal standards, 1.5 μg/mL PE (17:0/17:0), 7.5 μg/mL PG (17:0/17:0), 1.5 μg/mL PC (17:0/0:0), 0.30 μg/mL sphingosine (d17:1), 0.75 μg /mL ceramide (d18:1/17:0), 0.60 μg/mL SM (d18:1/17:0), 3 μg/ml palmitic acid-D3, 0.40 μg/mL PC (12:0/13:0), 30 μg/mL cholesterol-D7, 0.30 μg/mL TAG (17:0/17:1/17:0), 3.0 μg/mL DAG (12:0/12:0/0:0), 1.5 μg/mL DAG (18:1/2:0/0:0), 6.0 μg/mL MAG (17:0/0:0/0:0), 0.75 μg/mL PE (17:1/0:0), 15 μg/mL cholesteryl ester (22:1), and 75 ng/mL CUDA (12-[[(cyclohexylamino)carbonyl]amino]-dodecanoic acid. Each sample was sonicated for 30 s and vortexed for 10 s at room temperature.

The analytical conditions were based on the method of MS-DIAL. LC-MS measurement was performed on a LC system, Nexera X2 (Shimadzu) and TripleTOF 6600 equipped with DuoSpray ion source (Sciex). The injection volumes are 3 μL in positive mode and 5 μL in negative mode. The flow rate was 0.6 mL/min, and the temperature of the column oven was kept at 65 °C. The autosampler temperature was 4 °C. Mobile phase A was 6:4 CH_3_CN: H_2_O (v/v) containing 10 mM HCOONH_4_ and 0.1% formic acid. Mobile phase B was 9:1 isopropanol: CH_3_CN (v/v) containing 10 mM HCOONH_4_ and 0.1% formic acid. The LC method used Acquity UPLC CSH C18 column (Waters). Measurements were performed at the high sensitivity mode for TOF MS and product ion scan. SWATH (sequential window acquisition of all theoretical mass spectra) acquisition for both positive and negative ion mode was used to achieve data-independent acquisition. The same parameters for SWATH and the other MS system were used as described in the reference^75^. MS-DIAL version 3.98 software was used for the peak analysis and lipid annotation. The peak height values for each aligned peaks were exported for the statistical analysis using Metaboanalyst (https://www.metaboanalyst.ca/).

### DHA-rich diet

Male C57BL/6 J mice aged 8-week-old (~24 g) were obtained from Charles River. The basal diet was composed of the following components (g/kg diet): α-cornstarch, 542; casein, 184; sucrose, 92; corn oil (Nacalai Tesque, Kyoto, Japan) or DHA-rich oil (Nippon Suisan Kaisha, Tokyo, Japan), 92; cellulose, 46; AIN-93G mineral mixture, 32; AIN-93 vitamin mixture, 9.2; and L-cystine, 2.8. The concentrations of C22:6 n-3 (DHA) and C20:5 n-3 (EPA) in the DHA-rich oil were 58.0% and 7.1%, respectively. All animals were fed with the diet and water ad libitum.

### Statistics and reproducibility

All data are expressed as the mean ± S.E. Individual data points are shown in all graphs, with at least three independent biological replicates were used for each experiment. Two groups comparison was done using Student’s *t* test and the main effect in contraction force and fatigability data was analyzed using Two-way ANOVA. Relative expression was determined by comparing the treatment group to controls after normalization to controls. Statistical analyses were conducted using GraphPad Prism 7 software, with *p*-values < 0.05 are considered statistically significant as denoted in the figure legends. All reagents and resources used in this paper can be found in Supplementary Table 2.

### Reporting summary

Further information on research design is available in the Nature Portfolio Reporting Summary linked to this article.

### Supplementary information

Supplementary Information

Description of Additional Supplementary Files

Supplementary Data 1

Reporting Summary

## Data Availability

The raw DNA microarray data were deposited in the Gene Expression Omnibus (GEO) under accession GSE262199 (C57BL/6 mice: skeletal muscle-specific GDE5 (Gpcpd1)-deficient mice, GSM8159504 and GSM8159505). The metabolome and lipidome data were deposited in the DDBJ database with accession number PRJDB17952 (Project title, Skeletal muscle-specific glycerophosphodiester phosphodiesterase 5 (GDE5) deletion alter the lipidome and metabolome in the muscles, MTBKS236 and MTBKS237). The source data behind the graphs in the paper can be found in Supplementary Data 1. All data needed to evaluate the conclusions in the paper are present in the paper and/or the Supplementary Information.

## References

[1] Zeisel SH, Blusztajn JK (1994). Choline and human nutrition. Annu. Rev. Nutr..

[2] Li Z, Vance DE (2008). Phosphatidylcholine and choline homeostasis. J. Lipid. Res..

[3] Petersen EN, Chung H-W, Nayebosadri A, Hansen SB (2016). Kinetic disruption of lipid rafts is a mechanosensor for phospholipase D. Nat. Commun..

[4] Iorio E (2005). Alterations of choline phospholipid metabolism in ovarian tumor progression. Cancer Res..

[5] Corda D (2014). The emerging physiological roles of the glycerophosphodiesterase family. FEBS J.

[6] Yanaka N (2007). Mammalian glycerophosphodiester phosphodiesterases. Biosci. Biotechnol. Biochem..

[7] Okazaki Y (2010). A novel glycerophosphodiester phosphodiesterase, GDE5, controls skeletal muscle development via a non-enzymatic mechanism. J. Biol. Chem..

[8] Hoffman JR (2010). The effects of acute and prolonged CRAM supplementation on reaction time and subjective measures of focus and alertness in healthy college students. J. Int. Soc. Sports Nutr..

[9] Bellar D, LeBlanc NR, Campbell B (2015). The effect of 6 days of alpha glycerylphosphorylcholine on isometric strength. J. Int. Soc. Sports. Nutr..

[10] Sher RB (2006). A rostrocaudal muscular dystrophy caused by a defect in choline kinase beta, the first enzyme in phosphatidylcholine biosynthesis. J. Biol. Chem..

[11] Wu G, Sher RB, Cox GA, Vance DE (2010). Differential expression of choline kinase isoforms in skeletal muscle explains the phenotypic variability in the rostrocaudal muscular dystrophy mouse. Biochim. Biophys. Acta.

[12] Li Z (2006). The ratio of phosphatidylcholine to phosphatidylethanolamine influences membrane integrity and steatohepatitis. Cel. Metab..

[13] O’Leary EI, Jiang Z, Strub MP, Lee JC (2018). Effects of phosphatidylcholine membrane fluidity on the conformation and aggregation of N-terminally acetylated α-Synuclein. J. Biol. Chem..

[14] Ko M (2016). Phosphatidylcholine protects neurons from toxic effects of amyloid β-protein in culture. Brain Res..

[15] Mitchell TW (2010). The effect of exercise on the skeletal muscle phospholipidome of rats fed a high-fat diet. Int. J. Mol. Sci..

[16] Senoo N (2015). PGC-1α -mediated changes in phospholipid profiles of exercise-trained skeletal muscle. J. Lipid Res..

[17] Funai K (2016). Skeletal muscle phospholipid metabolism regulates insulin sensitivity and contractile function. Diabetes.

[18] Sakuma T, Nakade S, Sakane Y, Suzuki K-IT, Yamamoto T (2016). MMEJ-assisted gene knock-in using TALENs and CRISPR-Cas9 with the PITCh systems. Nat. Protoc..

[19] Gallazzini M, Ferraris JD, Burg MB (2008). GDPD5 is a glycerophosphocholine phosphodiesterase that osmotically regulates the osmoprotective organic osmolyte GPC. Proc. Natl. Acad. Sci. USA.

[20] Mugabo Y (2016). Identification of a mammalian glycerol-3-phosphate phosphatase: Role in metabolism and signaling in pancreatic β-cells and hepatocytes. Proc. Natl. Acad. Sci. USA.

[21] Irimia JM (2015). Muscle glycogen remodeling and glycogen phosphate metabolism following exhaustive exercise of wild type and laforin knockout mice. J. Biol. Chem..

[22] De Luca A, Pierno S, Camerino DC (2015). Taurine: the appeal of a safe amino acid for skeletal muscle disorders. J. Transl. Med..

[23] Uchitomi R (2019). Metabolomic analysis of skeletal muscle in aged mice. Sci. Rep..

[24] Gheller BJ (2021). Extracellular serine and glycine are required for mouse and human skeletal muscle stem and progenitor cell function. Mol. Metab..

[25] Martins-Bach AB, Bloise AC, Vainzof M, Rahnamaye Rabbani S (2012). Metabolic profile of dystrophic mdx mouse muscles analyzed with in vitro magnetic resonance spectroscopy (MRS). Magn. Reson. Imaging.

[26] Kohlwein SD, Veenhuis M, van der Klei IJ (2013). Lipid droplets and peroxisomes: key players in cellular lipid homeostasis or a matter of fat—store ’em up or burn ’em down. Genetics.

[27] Senoo N (2020). Glycerophospholipid profile alterations are associated with murine muscle‐wasting phenotype. Muscle Nerve.

[28] Tuazon MA, Henderson GC (2012). Fatty acid profile of skeletal muscle phospholipid is altered in mdx mice and is predictive of disease markers. Metabolism.

[29] Goto-Inoue N (2013). Lipidomics analysis revealed the phospholipid compositional changes in muscle by chronic exercise and high-fat diet. Sci. Rep..

[30] Andersson A, Sjödin A, Hedman A, Olsson R, Vessby B (2000). Fatty acid profile of skeletal muscle phospholipids in trained and untrained young men. Am. J. Physiol. Endocrinol..

[31] Senoo N (2021). Fasting increases 18:2-containing phosphatidylcholines to complement the decrease in 22:6-containing phosphatidylcholines in mouse skeletal muscle. PLoS ONE.

[32] Lands WEM (1960). Metabolism of glycerolipids. 2. The enzymatic acylation of lysolecithin. J. Biol. Chem..

[33] Fajardo VA (2015). Dietary docosahexaenoic acid supplementation reduces SERCA Ca2+ transport efficiency in rat skeletal muscle. Chem. Phys. Lipids.

[34] Goonasekera SA (2011). Mitigation of muscular dystrophy in mice by SERCA overexpression in skeletal muscle. J. Clin. Invest..

[35] Karatzaferi C, Chinn MK, Cooke R (2004). The force exerted by a muscle cross-bridge depends directly on the strength of the actomyosin bond. Biophys. J..

[36] Allen DG, Lamb GD, Westerblad H (2008). Skeletal muscle fatigue: cellular mechanisms. Physiol. Rev..

[37] Chin ER, Allen DG (1997). Effects of reduced muscle glycogen concentration on force, Ca2+ release and contractile protein function in intact mouse skeletal muscle. J. Physiol..

[38] Herrmann-Frank A, Lüttgau HC, Stephenson DG (1999). Caffeine and excitation-contraction coupling in skeletal muscle: a stimulating story. J. Muscle Res. Cell Motil..

[39] Andersson DC (2011). Ryanodine receptor oxidation causes intracellular calcium leak and muscle weakness in aging. Cell Metab..

[40] Harayama T, Riezman H (2018). Understanding the diversity of membrane lipid composition. Nat. Rev. Mol. Cell Biol..

[41] Shindou H, Shimizu T (2009). Acyl-CoA:lysophospholipid acyltransferases. J. Biol. Chem..

[42] Wang B, Tontonoz P (2019). Phospholipid remodeling in physiology and disease. Annu. Rev. Physiol..

[43] Kita Y, Shindou H, Shimizu T (2019). Cytosolic phospholipase A2 and lysophospholipid acyltransferases. Biochim. Biophys. Acta – Mol. Cell. Biol. Lipids..

[44] Fallbrook A, Turenne SD, Mamalias N, Kish SJ, Ross BM (1999). Phosphatidylcholine and phosphatidylethanolamine metabolites may regulate brain phospholipid catabolism via inhibition of lysophospholipase activity. Brain Res..

[45] Anaokar S (2019). The glycerophosphocholine acyltransferase Gpc1 is part of a phosphatidylcholine (PC)-remodeling pathway that alters PC species in yeast. J. Biol. Chem..

[46] Patton-Vogt J, de Kroon AIPM (2020). Phospholipid turnover and acyl chain remodeling in the yeast ER. Biochim. Biophys. Acta – Mol. Cell. Biol. Lipids..

[47] Ridgway, N. D. *Biochemistry of Lipids, Lipoproteins and Membranes* 209–236 (Elsevier, 2016).

[48] Valentine WJ (2022). Update and nomenclature proposal for mammalian lysophospholipid acyltransferases, which create membrane phospholipid diversity. J. Biol. Chem..

[49] Morgan T, Short F, Cobb L (1969). Effect of long-term exercise on skeletal muscle lipid composition. Am. J. Physiol..

[50] Helge JW, Ayre KJ, Hulbert AJ, Kiens B, Storlien LH (1999). Regular exercise modulates muscle membrane phospholipid profile in rats. J. Nutr..

[51] Le Guen M (2016). A 9-wk docosahexaenoic acid-enriched supplementation improves endurance exercise capacity and skeletal muscle mitochondrial function in adult rats. Am. J. Physiol. Endocrinol..

[52] Herbst EAF (2014). Omega-3 supplementation alters mitochondrial membrane composition and respiration kinetics in human skeletal muscle. J. Physiol..

[53] Henry R, Peoples GE, McLennan PL (2015). Muscle fatigue resistance in the rat hindlimb in vivo from low dietary intakes of tuna fish oil that selectively increase phospholipid n-3 docosahexaenoic acid according to muscle fibre type. Br. J. Nutr..

[54] Bergström J, Hermansen L, Hultman E, Saltin B (1967). Diet, muscle glycogen and physical performance. Acta Physiol. Scand..

[55] Hermansen L, Hultman E, Saltin B (1967). Muscle glycogen during prolonged severe exercise. Acta Physiol. Scand..

[56] Duhamel TA, Perco JG, Green HJ (2006). Manipulation of dietary carbohydrates after prolonged effort modifies muscle sarcoplasmic reticulum responses in exercising males. Am. J. Physiol. Regul. Integr. Comp. Physiol..

[57] Mancini D, Benaminovitz A, Cordisco ME, Karmally W, Weinberg A (1999). Slowed glycogen utilization enhances exercise endurance in patients with heart failure. J. Am. Coll. Cardiol..

[58] Ørtenblad N, Nielsen J (2015). Muscle glycogen and cell function - location, location, location. Scand. J. Med. Sci. Sports.

[59] Stephenson DG, Nguyen LT, Stephenson GMM (1999). Glycogen content and excitation‐contraction coupling in mechanically skinned muscle fibres of the cane toad. J. Physiol..

[60] Posterino GS, Lamb GD (2003). Effect of sarcoplasmic reticulum Ca2+ content on action potential-induced Ca2+ release in rat skeletal muscle fibres. J. Physiol..

[61] Bellinger AM (2009). Hypernitrosylated ryanodine receptor calcium release channels are leaky in dystrophic muscle. Nat. Med..

[62] Zalk R, Lehnart SE, Marks AR (2007). Modulation of the ryanodine receptor and intracellular calcium. Annu. Rev. Biochem..

[63] Belcastro AN (1993). Skeletal muscle calcium-activated neutral protease (calpain) with exercise. J. Appl. Physiol..

[64] Verburg E, Murphy RM, Stephenson DG, Lamb GD (2005). Disruption of excitation-contraction coupling and titin by endogenous Ca2+-activated proteases in toad muscle fibres. J. Physiol..

[65] Zhang B-T, Yeung SS, Allen DG, Qin L, Yeung EW (2008). Role of the calcium-calpain pathway in cytoskeletal damage after eccentric contractions. J. Appl. Physiol..

[66] Sreter FA (1970). Effect of denervation on fragmented sarcoplasmic reticulum of white and red muscle. Exp. Neurol..

[67] Midrio M (2006). The denervated muscle: facts and hypotheses. A historical review. Eur. J. Appl. Physiol..

[68] Borkman M (1993). The Relation between insulin sensitivity and the fatty-acid composition of skeletal-muscle phospholipids. N. Engl. J. Med..

[69] Clore JN (2000). Changes in phsophatidylcholine fatty acid composition are associated with altered skeletal muscle insulin responsiveness in normal man. Metabolism.

[70] Jensen J (2006). Muscle glycogen inharmoniously regulates glycogen synthase activity, glucose uptake, and proximal insulin signaling. Am. J. Physiol. Endocrinol..

[71] Kim Y-B (2005). Muscle-specific deletion of the glut4 glucose transporter alters multiple regulatory steps in glycogen metabolism. Mol. Cell. Biol..

[72] Nakagawa Y (2016). Ultra-superovulation for the CRISPR-Cas9-mediated production of gene-knockout, single-amino-acid-substituted, and floxed mice. Biol. Open..

[73] Nitschke F (2017). Abnormal glycogen chain length pattern, not hyperphosphorylation, is critical in Lafora disease. EMBO Mol. Med..

[74] Nitschke F (2013). Hyperphosphorylation of glucosyl C6 carbons and altered structure of glycogen in the neurodegenerative epilepsy lafora disease. Cell Metab..

[75] Tsugawa H (2015). MS-DIAL: data-independent MS/MS deconvolution for comprehensive metabolome analysis. Nat. Methods..

