# Supplementary material for: GDE5/Gpcpd1 activity determines phosphatidylcholine composition in skeletal muscle and regulates contractile force in mice
Source: Commun Biol. 2024 May 20;7:604. doi: 10.1038/s42003-024-06298-z (PMC11106330; doi:10.1038/s42003-024-06298-z)
Supplement: Supplementary file 1 — Supplementary Information [file 42003_2024_6298_MOESM1_ESM.docx]

GDE5 activity determines phosphatidylcholine composition in skeletal muscle and regulates contractile force in mice

Rahmawati Aisyah, Noriyasu Ohshima, Daiki Watanabe, Yoshiko Nakagawa, Tetsushi Sakuma, Felix Nitschke, Minako Nakamura, Koji Sato, Kaori Nakahata, Chihiro Yokoyama, Charlotte R. Marchioni, Thanutchaporn Kumrungsee, Takahiko Shimizu, Yusuke Sotomaru, Toru Takeo, Naomi Nakagata, Takashi Izumi, Shinji Miura, Berge A Minassian, Takashi Yamamoto, Masanobu Wada, and Noriyuki Yanaka

*To whom correspondence should be addressed: Noriyuki Yanaka, Graduate School of Integrated Sciences for Life, Hiroshima University, Higashi-Hiroshima, 739-8528, Japan. Tel.: +81-82-4247979; Fax: +81-82-4247916; E-mail: yanaka@hiroshima-u.ac.jp

Supplementary Figures:

Supplementary Figure S1

Supplementary Figure S2

Supplementary Figure S3

Supplementary Figure S4

Supplementary Figure S5

Supplementary Figure S6

Supplementary Figure S7

Supplementary Figure S8

Supplementary Figure S9

Supplementary Figure S10

Supplementary Figure S11

Supplementary Tables:

Supplementary Table 1

Supplementary Table 2

**
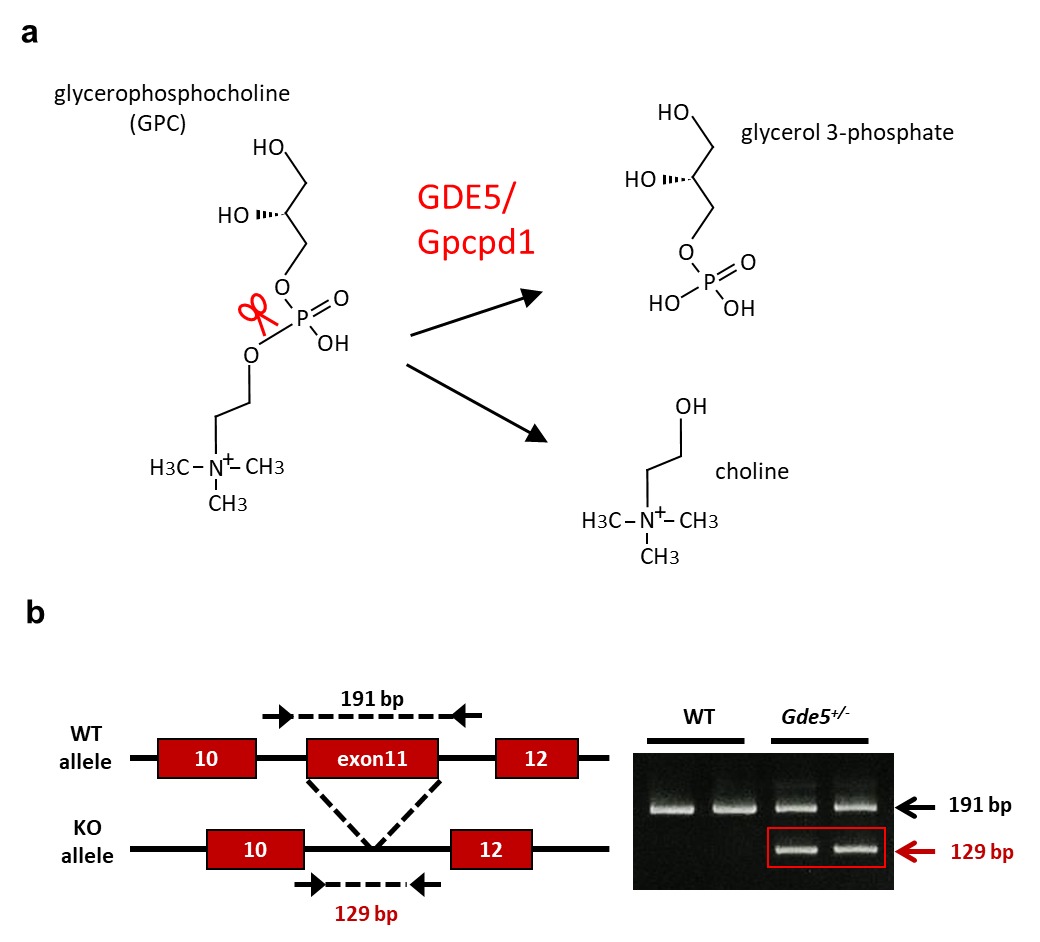
**

**Figure S1, related to Figure 1. Generation of whole-body *Gde5* (*Gpcpd1*) knockout mice.**

(a) GDE5/Gpcpd1 hydrolyzes glycerophosphocholine (GPC) into choline and glycerol 3-phosphate.

(b) Gene targeting of the GDE5 gene. PCR genotyping using tail DNA, amplifying 191-bp and 129-bp products for the WT and KO alleles, respectively.

**
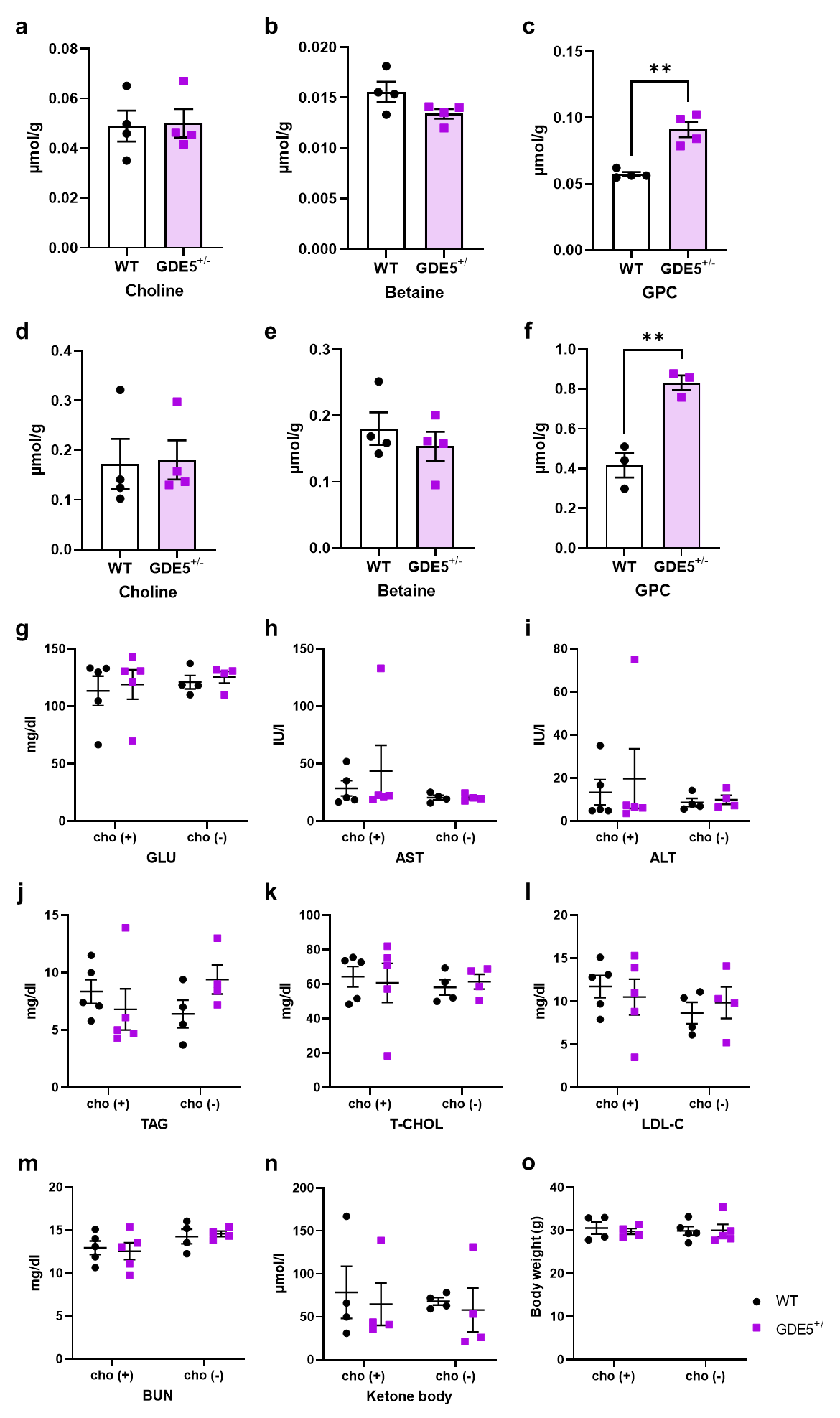
**

**Figure S2, related to Figure 1. Choline metabolites and serum profiles in *Gde5^+/-^* and WT mice under choline deficiency.**

(a-c) Choline metabolites level in skeletal muscle of *Gde5^+/-^* and WT mice under choline deficiency diet.

(d-f) Choline metabolites level in liver of *Gde5^+/-^* and WT mice under choline deficiency diet.

(g) Glucose level of *Gde5^+/-^* and WT mice under choline deficiency diet.

(h) AST level of *Gde5^+/-^* and WT mice under choline deficiency diet.

(i) ALT level of *Gde5^+/-^* and WT mice under choline deficiency diet.

(j) TAG level of *Gde5^+/-^* and WT mice under choline deficiency diet.

(k) Total cholesterol level of *Gde5^+/-^* and WT mice under choline deficiency diet.

(l) LDL-cholesterol level of *Gde5^+/-^* and WT mice under choline deficiency diet.

(m) Blood urea nitrogen level of *Gde5^+/-^* and WT mice under choline deficiency diet.

(n) Ketone body of *Gde5^+/-^* and WT mice under choline deficiency diet.

(o) Body weight of *Gde5^+/-^* and WT mice under choline deficiency diet.

Values are means ± SEM. Statistical analysis was performed with Student’s t test. **p < 0.01.

**
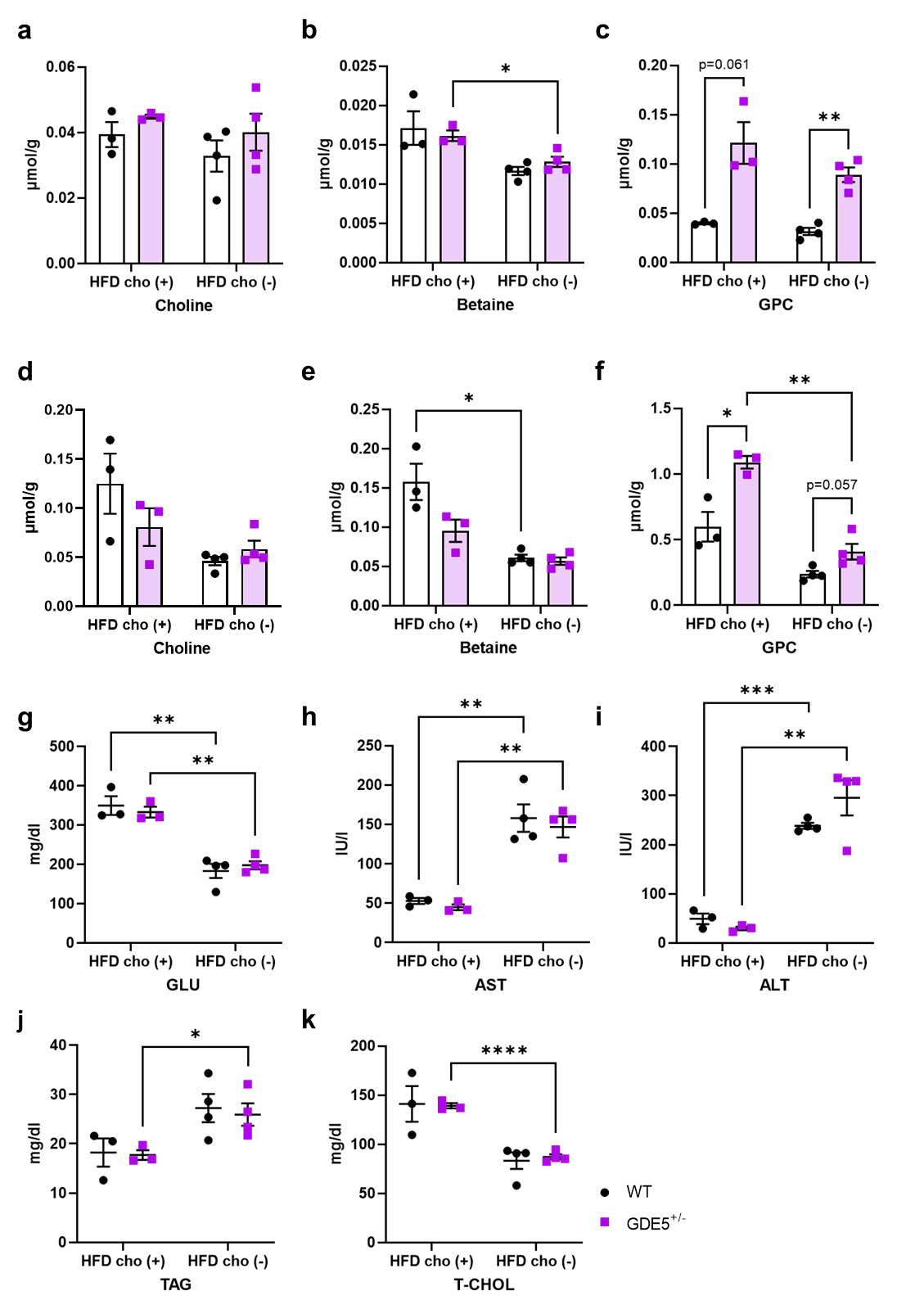
**

**Figure S3, related to Figure 1. Choline metabolites and serum profiles in *Gde5^+/-^* and WT mice under high-fat diet (HFD) and choline-deficient, high-fat diet (CD-HFD).**

(a-c) Choline metabolites level in skeletal muscle of *Gde5^+/-^* and WT mice under HFD and CD-HFD.

(d-f) Choline metabolites level in liver of *Gde5^+/-^* and WT mice under HFD and CD-HFD.

(g) Glucose level of *Gde5^+/-^* and WT mice under HFD and CD-HFD.

(h) AST level of *Gde5^+/-^* and WT mice under HFD and CD-HFD.

(i) ALT level of *Gde5^+/-^* and WT mice under HFD and CD-HFD.

(j) TAG level of *Gde5^+/-^* and WT mice under HFD and CD-HFD.

(k) Total cholesterol level of *Gde5^+/-^* and WT mice under HFD and CD-HFD.

Values are means ± SEM. Statistical analysis was performed with Student’s t test. **p < 0.01.

**
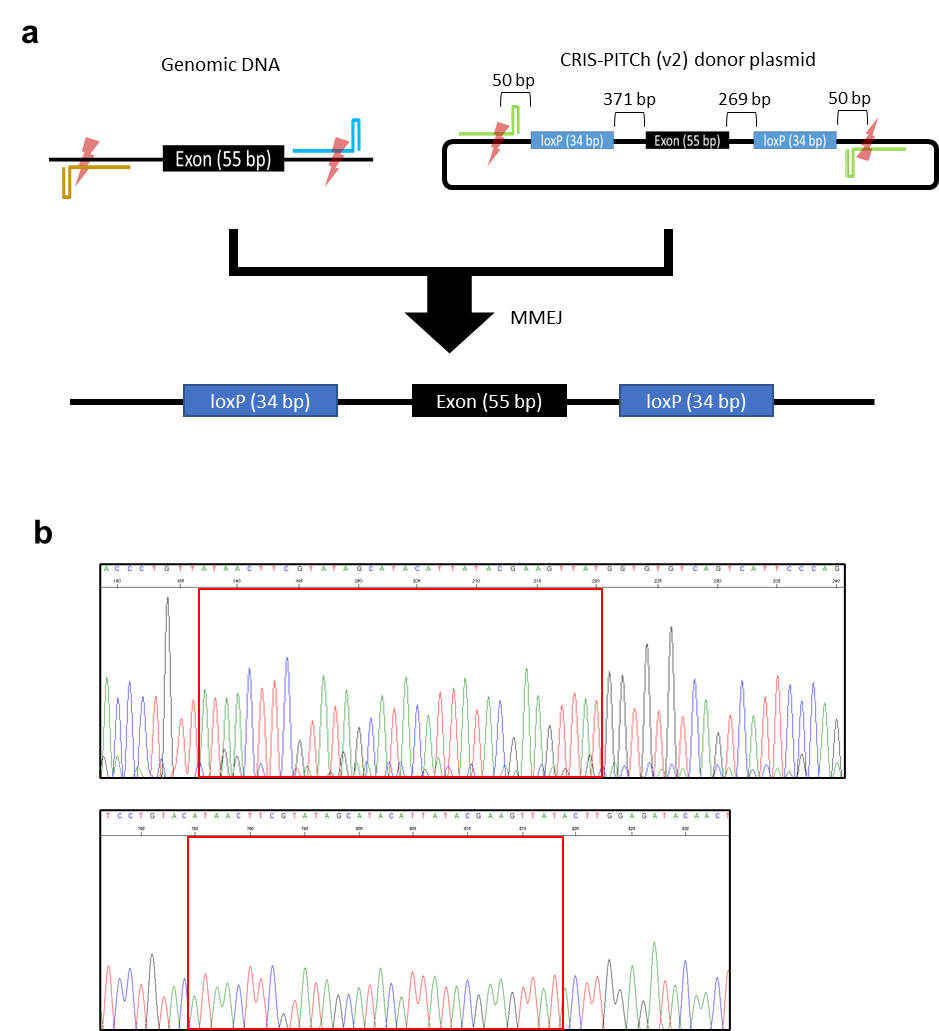
**

**Figure S4.** **Generation of floxed mice at the *Gde5* locus.**

(a) Schematic illustration to generate a floxed allele at the *Gde5* locus, mediated by the CRIS-PITCh (v2) system. Two gene-specific gRNAs were designed upstream and downstream of exon 11. A PITCh donor plasmid was designed to carry two loxP sites flanking exon 11.

(b) Sequence data (sense strand) of upstream and downstream of exon 11. Red box shows the loxP sequence located in the introns flanking exon 11 of the *Gde5* gene.

**
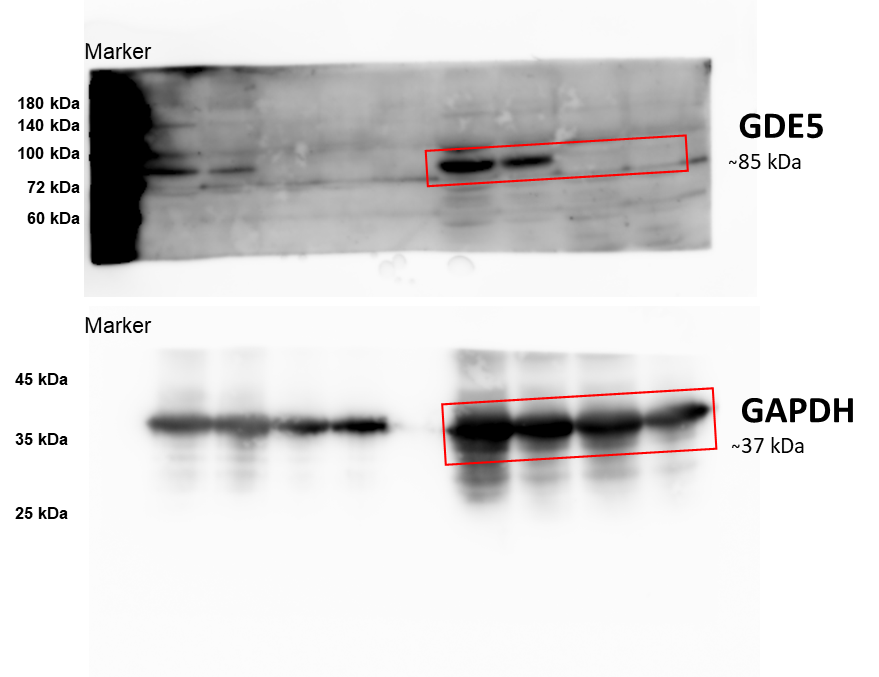
**

**Figure S5, related to Figure 2. Uncropped western blotting pictures of GDE5**

GDE5 protein expression in the skeletal muscle of *Gde5* skKO and WT mice (original blot of Fig. 2A)

**
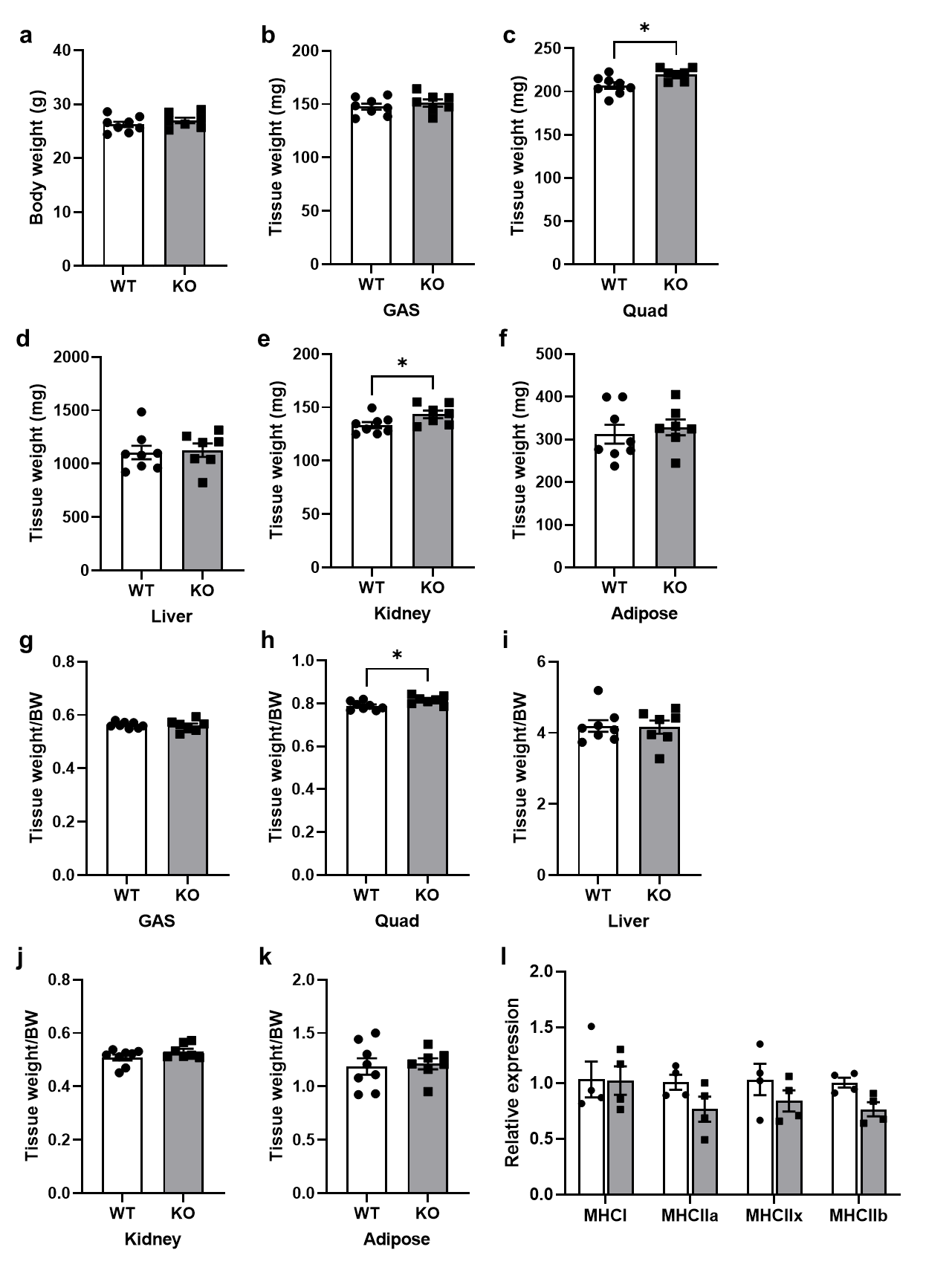
Figure S6, related to Figure 2. Body weight, tissue weight, and muscle types in *Gde5* skKO and WT mice.**

(a) Body weight of *Gde5* skKO and WT mice.

(b-f) Various tissue weights of *Gde5* skKO and WT mice, specifically gastrocnemius muscle (b), quadriceps muscle (c), liver (d), kidney (e), and adipose (f).

(g-k) Various tissue weights compared to body weight of *Gde5* skKO and WT mice, specifically gastrocnemius muscle (g), quadriceps muscle (h), liver (i), kidney (j), and adipose (k).

(l) qPCR analysis of muscle fiber type in *Gde5* skKO and WT mice.

Values are means ± SEM. Statistical analysis was performed with Student’s t test. *p < 0.05.

**
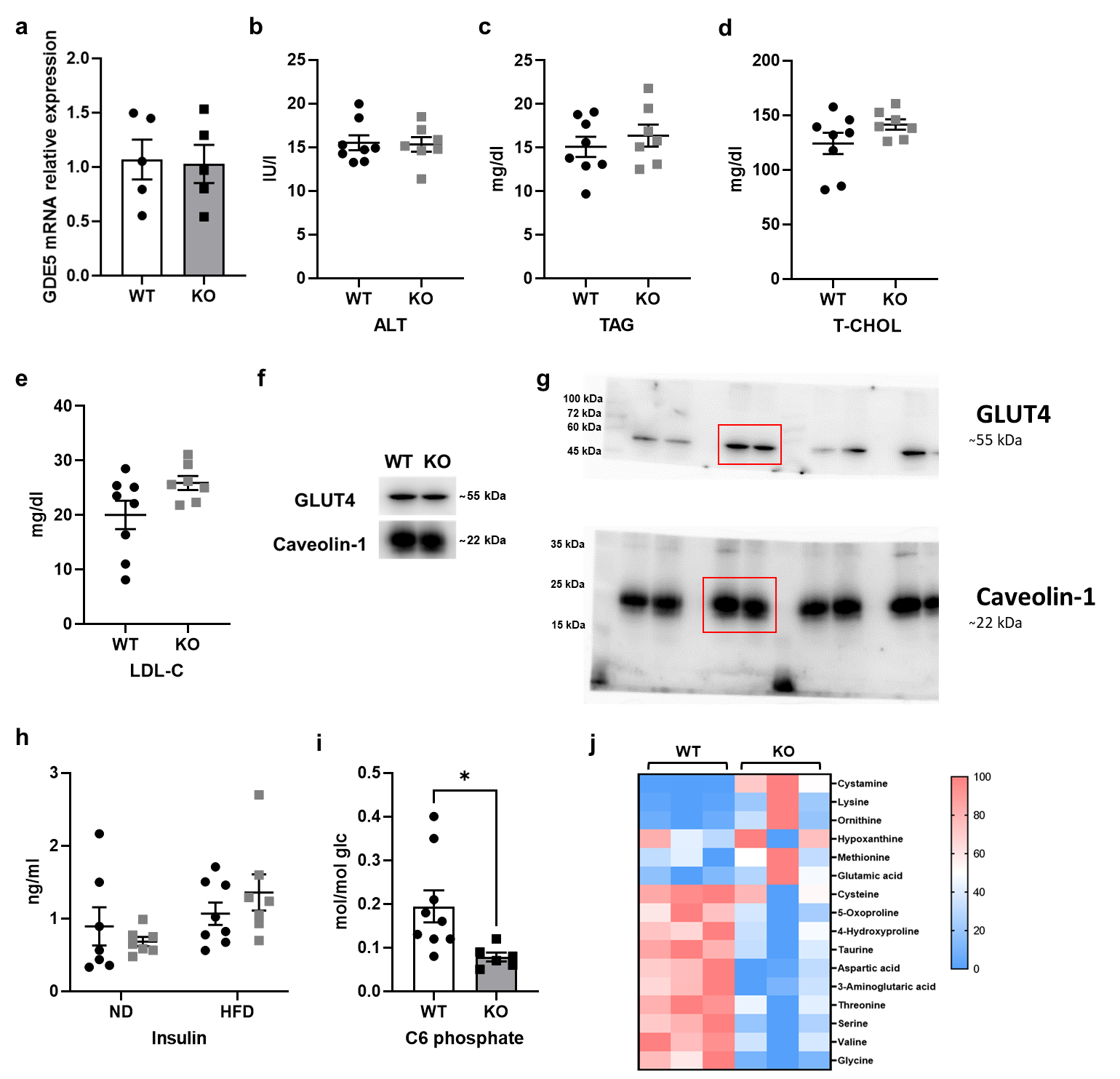
**

**Figure S7, related to Figure 2 and 3. *Gde5* mRNA expression, serum profiles under HFD, GLUT4 expression, glycogen C6 phosphate, and amino acid level in skeletal muscles of *Gde5* skKO and WT.**

(a) *Gde5* mRNA expression in the liver of *Gde5* skKO and WT mice.

(b) ALT level in in *Gde5* skKO and WT mice under HFD.

(c) TAG level in *Gde5* skKO and WT mice under HFD.

(d) Total cholesterol level in *Gde5* skKO and WT mice under HFD.

(e) LDL-cholesterol level in *Gde5* skKO and WT mice under HFD.

(f) Western blot representative of GLUT4 expression in total membrane protein isolated from skeletal muscle of *Gde5* skKO and WT mice.

(g) GLUT4 protein expression in total membrane protein isolated from skeletal muscle of *Gde5* skKO and WT mice (original blot of Fig. S7f)

(h) Blood insulin level under chow and HFD in *Gde5* skKO and WT mice.

(i) C6 phosphate level in skeletal muscle of *Gde5* skKO and WT mice fed with control chow diet.

(j) Heatmap representation of amino acid and its related metabolites level in *Gde5* skKO and WT mice.

Values are means ± SEM. Statistical analysis was performed with Student’s t test. *p < 0.05.

**
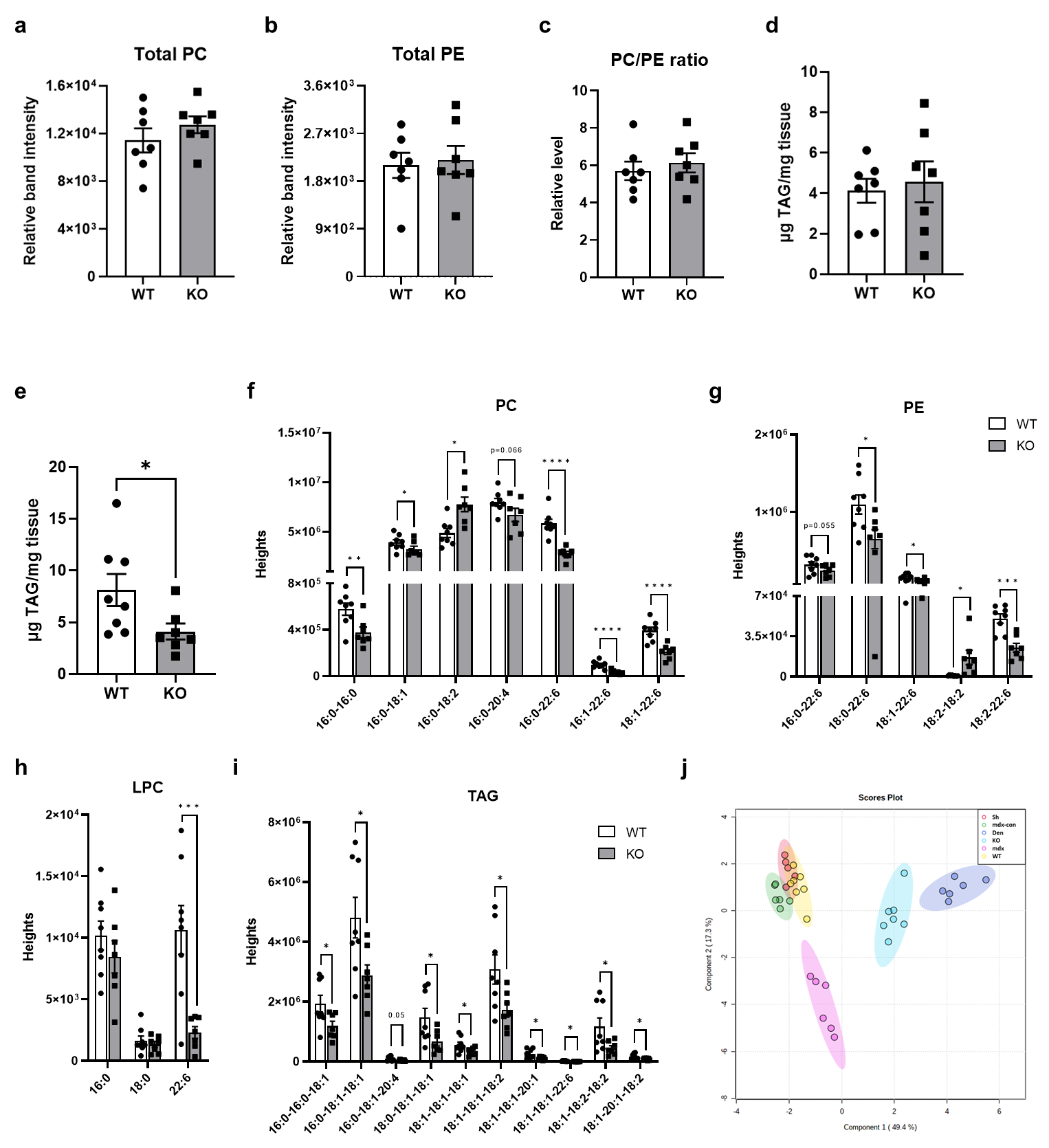
**

**Figure S8, related to Figure 4. Lipid profiles in skeletal muscle of *Gde5* skKO and WT mice.**

(a) Total PC level in skeletal muscle of *Gde5* skKO and WT mice under normal chow diet.

(b) Total PE level in skeletal muscle of *Gde5* skKO and WT mice under normal chow diet.

(c) PC/PE ratio in skeletal muscle of *Gde5* skKO and WT mice under normal chow diet.

(d) Total TAG content in *Gde5* skKO and WT muscle under normal chow diet.

(e) Total TAG content in *Gde5* skKO and WT muscle under HFD.

(f) PC molecular species in *Gde5* skKO and WT muscle under HFD.

(g) PE molecular species in *Gde5* skKO and WT muscle under HFD.

(h) LPC molecular species in *Gde5* skKO and WT muscle under HFD.

(i) TAG molecular species in *Gde5* skKO and WT muscle under HFD.

(j) Clustering of phospholipid composition between *Gde5* skKO, denervation, and mdx muscle.

Values are means ± SEM. Statistical analysis was performed with Student’s t test. *p < 0.05.

**
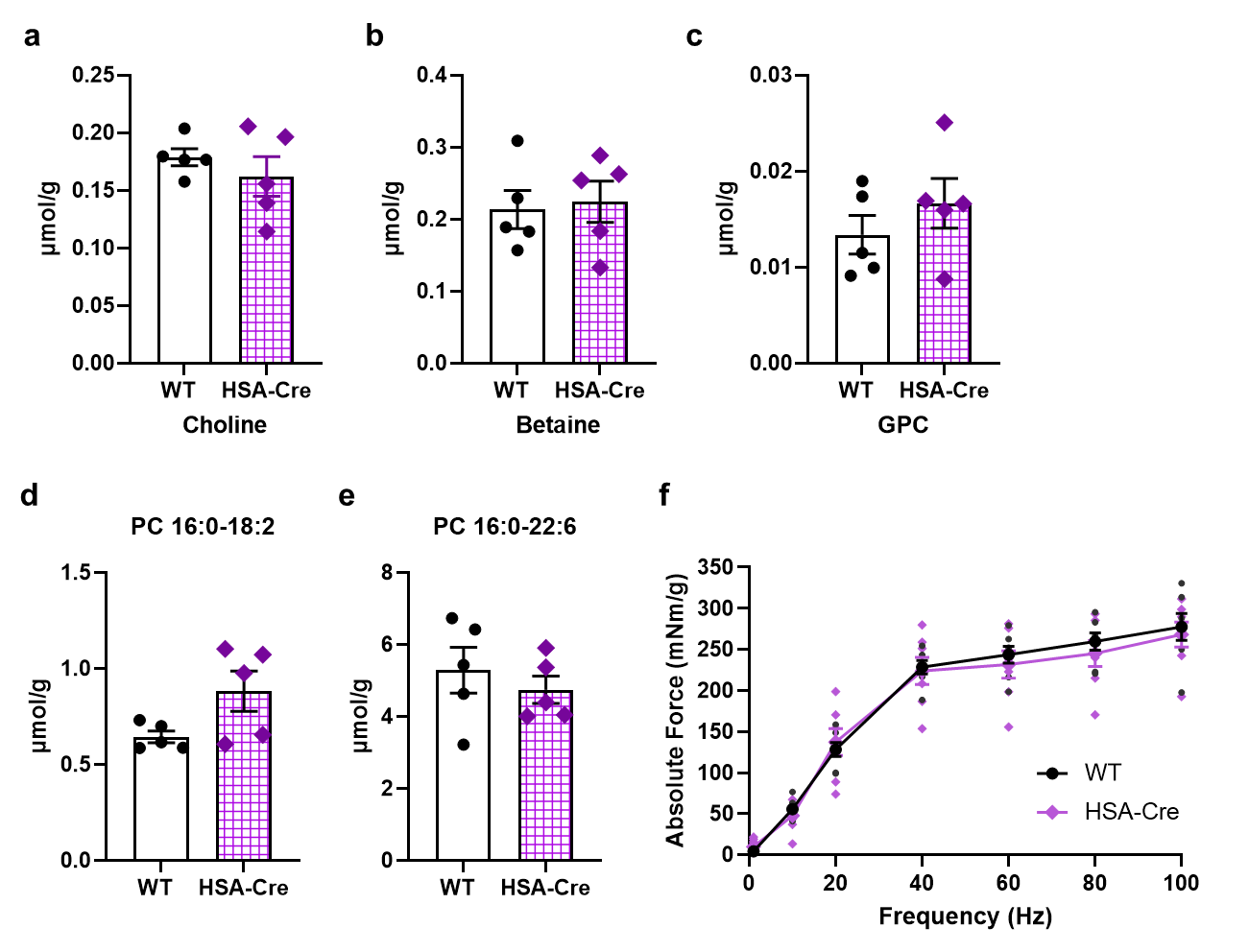
**

**Figure S9, related to Figure 4. Choline metabolites, PC composition, and contractile force in HSA-Cre mice compared to WT mice.**

(a-c) Choline metabolites level in skeletal muscle of HSA-Cre and WT mice under normal chow diet.

(d-e) PC levels of HSA-Cre and WT mice under normal chow diet.

(f) Contractile force test of HSA-Cre and WT mice under normal chow diet.

Values are means ± SEM.

**
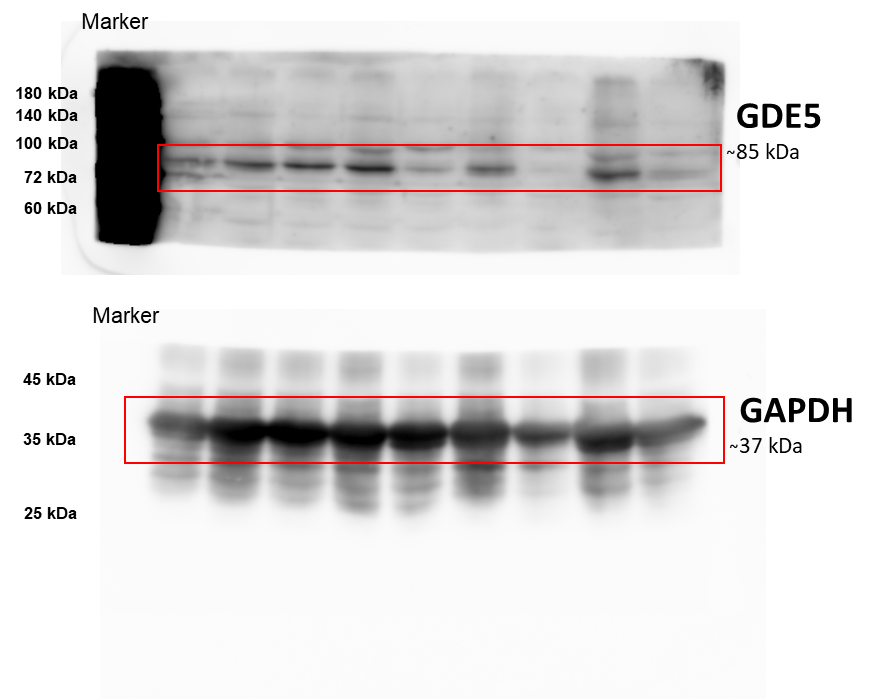
**

**Figure S10, related to Figure 5. Uncropped western blotting pictures of GDE5**

GDE5 protein expression in the skeletal muscle of denervated and mdx mice compared to their respective controls (original blot of Fig. 5f)

**
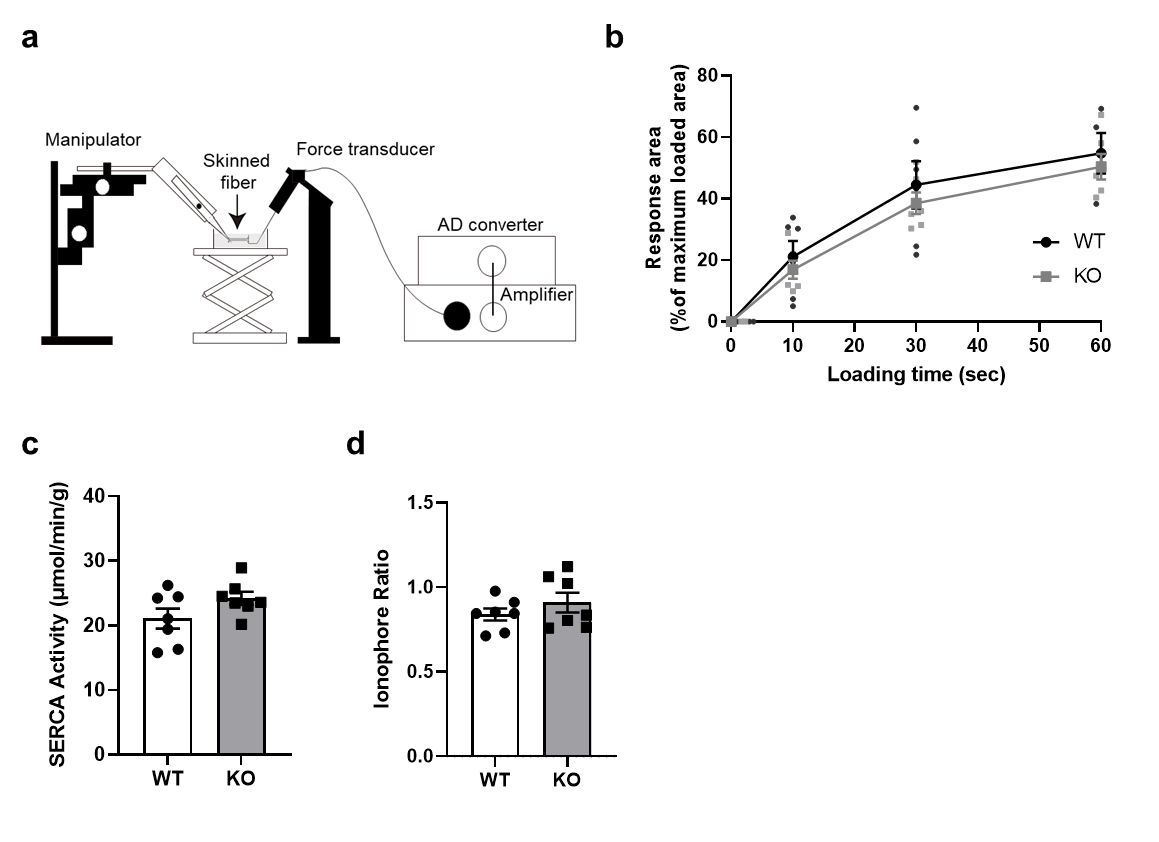
**

**Figure S11, related to Figure 7. Biochemical and skinned fiber analysis of *Gde5* skKO and WT muscle.**

(a) Schematic figure of skinned fiber experiment.

(b) Load release response of *Gde5* skKO and WT skinned fiber.

(c) SERCA activity assay of *Gde5* skKO and WT muscle.

(d) Ionophore ratio of *Gde5* skKO and WT muscle.

Values are means ± SEM.

**Supplementary Table 1. Primer sequences for qPCR**

| Oligonucleotides | Primer Sequence (5'→3') |
| --- | --- |
| Mouse *HSA-Cre* Forward: | AAATACTCTGAGAGTCCAAACCGGGCCCC |
| Mouse *HSA-Cre* Reverse: | CAGTGGGTTCGAACGCTAGAGCCTGTT |
| Mouse *Gde5* Forward: | ATTGGACGTTGGACATCGTG |
| Mouse *Gde5* Reverse: | GAGGTCATGATACACCACGG |
| Mouse *Flox* Forward: | GGGCATTCAATCCCATTACAG |
| Mouse *Flox* Reverse: | CCTCCAAATGACTTCATACTGGC |
| Mouse *Gck* Forward: | AGAGCAGATCCTGGCAGAGT |
| Mouse *Gck* Reverse: | TGGTTCCTCCCAGGTCTAAG |
| Mouse *Myh7* Forward | ACTGTCAACACTAAGAGGGTC |
| Mouse *Myh7* Reverse | TTGGATTTGATCTTCCAGGG |
| Mouse *Myh2* Forward | AAGCGAAGAGTAAGGCTGTC |
| Mouse *Myh2* Reverse | GTGATTGCTTGCAAAGGAAC |
| Mouse *Myh1* Forward | CCAAGTGCAGGAAAGTGACC |
| Mouse *Myh1* Reverse | AGGAAGAGACTGACGAGCTC |
| Mouse *Myh4* Forward | ACAAGCTGCGGGTGAAGAGC |
| Mouse *Myh4* Reverse | CAGGACAGTGACAAAGAACG |

**Supplementary Table 2. Reagents and resources**

| REAGENT or RESOURCE | SOURCE | IDENTIFIER |
| --- | --- | --- |
| Antibodies |  |  |
| Rabbit polyclonal anti-GDE5 | Okazaki et al., 2010 | N/A |
| Mouse monoclonal anti-GAPDH | Fujifilm WAKO | 016-25523 |
| Mouse monoclonal anti-GLUT4 | Proteintech | 66846-1-lg |
| Rabbit polyclonal anti-caveolin-1 | Transduction Laboratories | c13630 |
| Chemicals, peptides, and recombinant proteins | | |
| Isoflurane | Fujifilm WAKO | 099-06571 |
| Insulin | Eli Lilly Japan | 2492403A4051 |
| Glucose | Fujifilm WAKO | 047-31161 |
| Hexokinase | Fujifilm WAKO | 308-50681 |
| Glucose-6-phosphate dehydrogenase | Fujifilm WAKO | 303-50153 |
| Lactate dehydrogenase | Fujifilm WAKO | 300-52721 |
| Pyruvate kinase | Fujifilm WAKO | 151999 (5k U) |
| Medetomidine | Orion Pharma Animal Health | 86347-15-1 |
| Midazolam | Sandoz | 614243022 |
| Butorphanol tartrate | Meiji Seika Pharma | N/A |
| 2-isopropylmalic acid | Sigma-Aldrich | 333115 |
| *N*-methyl-*N*-trimethylsilyl-trifluoroacetamide | TCI | M0672 |
| Methoxyamine hydrochloride | SUPELCO | 33045-U |
| MgCl_2_ | Nacalai Tesque | 20935-05 |
| Adenosine-5'-triphosphate disodium salt hydrate (ATP) | Nacalai Tesque | 01072-11 |
| β-Nicotinamide adenine dinucleotide phosphate sodium salt (NADP) | Nacalai Tesque | 24336-64 |
| Sucrose | Nacalai Tesque | 30406-25 |
| Malate | Nacalai Tesque | 21101-72 |
| Phenylmethanesulfonyl fluoride | Nacalai Tesque | 27327-81 |
| Pepstatin | Nacalai Tesque | 26436-52 |
| Leupeptin | Nacalai Tesque | 43449-62 |
| Benzamidine | Nacalai Tesque | 01937-91 |
| HEPES | Nacalai Tesque | 17514-86 |
| KCl | Sigma-Aldrich | 1.04938.0500 |
| Sodium Azide | Sigma-Aldrich | S2002-100G |
| Ethylene glycol bis (β-aminoethylether)-*N*,*N*,*N'*,*N'*-tetraacetic acid (EGTA) | Nacalai Tesque | 15214-21 |
| Phosphoenolpyruvate | Sigma-Aldrich | P7262-5G |
| Calcium ionophore | Sigma-Aldrich | C7522-1MG |
| NADH | Sigma-Aldrich | N8129-100MG |
| CaCl_2_ | Nacalai Tesque | 06729-55 |
| Amyloglucosidase | Megazyme | E-AMGDF-40ML |
| Glucose-6-phosphate dehydrogenase from Leuconostoc | Sigma-Aldrich | G5760 |
| Glucose-6-phosphate dehydrogenase from Leuconostoc | Roche | 1016587001 |
| Hexokinase from yeast | Roche | 11426362001 |
| Fetal bovine serum | Wisent | 095150 |
| 3-(4,5-dimethylthiazol-2-yl)-2,5-diphenyltetrazolium | Sigma-Aldrich | M2128 |
| Phenazine-methosulfate | Sigma-Aldrich | P9625 |
| Nicotinamide adenine dinucleotide phosphate (NADP) | Roche | 10128058001 |
| Glucose-6-phosphate (G6P) | Sigma-Aldrich | G7879 |
| Disodium dihydrogen ethylenediamine-tetraacetate dihydrate (EDTA) | Nacalai Tesque | 15111-32 |
| Glutathione (Reduced Form) | Fujifilm WAKO | 077-02011 (1 g) |
| Formic acid | Merck | 5.33002.0050 |
| Aprotinin | Nacalai Tesque | 03346-84 |
| Phosphatase inhibitor cocktail | Nacalai Tesque | 07575-51 |
| Protease inhibitor cocktail | Fujifilm WAKO | 163-2601 |
| sodium dodecyl sulfate (SDS) | Nacalai Tesque | 31607-65 |
| Glycerol | Nacalai Tesque | 17017-35 |
| Methanol | Nacalai Tesque | 21929-23 |
| Skim milk | Nacalai Tesque | 31149-75 |
| Chloroform | Fujifilm WAKO | 038-02601 |
| Acetonitrile | Sigma-Aldrich | 1.14291.4000 |
| Isopropanol | Nacalai Tesque | 29113-53 |
| Toluene | Fujifilm WAKO | 204-01861 |
| Choline-tri(methyl-d3) chloride | Sigma-Aldrich | 492051 |
| *N*-(Carboxymethyl)-*N*,*N*,*N*-trimethyl-d9-ammonium chloride | CDN Isotopes | D3352 |
| sn-Glycero-3-phosphocholine-d9 | Cayman Chemical | 26099 |
| PC 17:0-17:0 | Avanti | 830456X |
| Critical commercial assays |  |  |
| *DC* Protein assay kit | BioRad Laboratories | 5000112 |
| Triglyceride E-Test Wako | Fujifilm WAKO | 432-40201 |
| RNAeasy micro kit | Qiagen | 74004 |
| THUNDERBIRD® SYBR® qPCR Mix | TOYOBO | QPS-201 |
| EnzCheck® Phospholipase A_2_ assay kit | Invitrogen | E10217 |
| Recombinant DNA |  |  |
| pTA2 vector | TOYOBO | TAK-201 |
| pCRIS-PITChv2-FBL | Sakuma et al., 2016 | N/A |
| Resource |  |  |
| Mouse: Gde5^+/−^ | Nakagawa et al., 2016 | N/A |
| Mouse: Gde5-floxed (*Gde5*^flox/flox^) | This paper | N/A |
| Mouse: HSA–Cre transgenic | Miniou et al., 1999 | N/A |
| Mouse: Duchenne muscular dystrophy mouse models *Mdx* | Senoo et al., 2020 | N/A |
| Software and algorithms |  |  |
| MassLynx version 4.1 | Waters | N/A |
| Agilent Feature Extraction Program version 9.5 | Agilent | N/A |
| GCMS solution software version 4.41 | Shimazu | N/A |
| MS-DIAL version 3.98 | http://prime.psc.riken.jp/compms/msdial/main.html | N/A |
| GraphPad Prism 9 | GraphPad | N/A |
| Lab-Chart version 7 | ADInstruments | N/A |
