# Supplementary material for: GDE5/Gpcpd1 activity determines phosphatidylcholine composition in skeletal muscle and regulates contractile force in mice
Source: Commun Biol. 2024 May 20;7:604. doi: 10.1038/s42003-024-06298-z (PMC11106330; doi:10.1038/s42003-024-06298-z)
Supplement: Supplementary file 2 — Description of Additional Supplementary Files [file 42003_2024_6298_MOESM2_ESM.docx]

**Description of Additional Supplementary Files**

**File name**: Supplementary Data 1
**Description:** The source data behind the graphs in the paper.
