# Supplementary material for: GDE5/Gpcpd1 activity determines phosphatidylcholine composition in skeletal muscle and regulates contractile force in mice
Source: Commun Biol. 2024 May 20;7:604. doi: 10.1038/s42003-024-06298-z (PMC11106330; doi:10.1038/s42003-024-06298-z)
Supplement: Supplementary file 4 — Reporting Summary [file 42003_2024_6298_MOESM4_ESM.pdf]

nature portfolio

Corresponding author(s): Noriyuki Yanaka

Last updated by author(s): Apr 1, 2024

Reporting Summary

Nature Portfolio wishes to improve the reproducibility of the work that we publish. This form provides structure for consistency and transparency in reporting. For further information on Nature Portfolio policies, see our Editorial Policies and the Editorial Policy Checklist.

Statistics

For all statistical analyses, confirm that the following items are present in the figure legend, table legend, main text, or Methods section.

n/a	Confirmed	
☐	☒	The exact sample size (*n*) for each experimental group/condition, given as a discrete number and unit of measurement
☐	☒	A statement on whether measurements were taken from distinct samples or whether the same sample was measured repeatedly
☐	☒	The statistical test(s) used AND whether they are one- or two-sided<br>*Only common tests should be described solely by name; describe more complex techniques in the Methods section.*
☒	☐	A description of all covariates tested
☒	☐	A description of any assumptions or corrections, such as tests of normality and adjustment for multiple comparisons
☐	☒	A full description of the statistical parameters including central tendency (e.g. means) or other basic estimates (e.g. regression coefficient) AND variation (e.g. standard deviation) or associated estimates of uncertainty (e.g. confidence intervals)
☐	☒	For null hypothesis testing, the test statistic (e.g. *F*, *t*, *r*) with confidence intervals, effect sizes, degrees of freedom and *P* value noted<br>*Give P values as exact values whenever suitable.*
☒	☐	For Bayesian analysis, information on the choice of priors and Markov chain Monte Carlo settings
☒	☐	For hierarchical and complex designs, identification of the appropriate level for tests and full reporting of outcomes
☒	☐	Estimates of effect sizes (e.g. Cohen's *d*, Pearson's *r*), indicating how they were calculated

Our web collection on statistics for biologists contains articles on many of the points above.

Software and code

Policy information about availability of computer code

Data collection	GC/MS analysis data was collected based on Smart Metabolites Database Release 3.01 (Shimadzu, Japan) that contains the data acquisition parameters for 467 compounds in multiple reaction monitoring (MRM) mode. Lipidomic analysis data was acquired on SWATH (sequential window acquisition of all theoretical mass spectra) acquisition for both positive and negative ion mode. MS-DIAL version 3.98 software was used for the peak detection, peak alignment, and lipid annotation. LC/MS data was acquired on MassLynx version 4.1 (Waters). DNA microarray data were obtained using Agilent Feature Extraction software (Agilent Technologies).
Data analysis	GraphPad Prism (version 9) was used for data visualizaion and for statistical tests. LC/MS data was analyzed on QuanLynx (Waters). Contractile force data was analyzed using Lab-Chart software (version 7, ADInstruments, Japan). DNA microarray data was analyzed by the Agilent Feature Extraction Program (version 9.5).

For manuscripts utilizing custom algorithms or software that are central to the research but not yet described in published literature, software must be made available to editors and reviewers. We strongly encourage code deposition in a community repository (e.g. GitHub). See the Nature Portfolio guidelines for submitting code & software for further information.

Data

Policy information about availability of data

All manuscripts must include a data availability statement. This statement should provide the following information, where applicable:

- Accession codes, unique identifiers, or web links for publicly available datasets
- A description of any restrictions on data availability
- For clinical datasets or third party data, please ensure that the statement adheres to our policy

Materials used in this study will be made available under an appropriate Material Transfer Agreement. All the data are provided in the paper.

Research involving human participants, their data, or biological material

Policy information about studies with human participants or human data. See also policy information about sex, gender (identity/presentation), and sexual orientation and race, ethnicity and racism.

Reporting on sex and gender	n/a
Reporting on race, ethnicity, or other socially relevant groupings	n/a
Population characteristics	n/a
Recruitment	n/a
Ethics oversight	n/a

Note that full information on the approval of the study protocol must also be provided in the manuscript.

Field-specific reporting

Please select the one below that is the best fit for your research. If you are not sure, read the appropriate sections before making your selection.

☒ Life sciences ☐ Behavioural & social sciences ☐ Ecological, evolutionary & environmental sciences

For a reference copy of the document with all sections, see nature.com/documents/nr-reporting-summary-flat.pdf

Life sciences study design

All studies must disclose on these points even when the disclosure is negative.

Sample size	We used sufficient sample sizes for comparisons between groups for these experiments.
Data exclusions	No data were excluded.
Replication	The key results, lipid and metabolites analysis and contraction force analysis, were repeated twice independently in technical triplicate with similar results.
Randomization	This is an observational study, randamization is not relevant.
Blinding	This is an observational study, investigators were not blinded.

Reporting for specific materials, systems and methods

We require information from authors about some types of materials, experimental systems and methods used in many studies. Here, indicate whether each material, system or method listed is relevant to your study. If you are not sure if a list item applies to your research, read the appropriate section before selecting a response.

Materials & experimental systems

n/a	Involved in the study
	☒ Antibodies
☒	☐ Eukaryotic cell lines
☒	☐ Palaeontology and archaeology
	☒ Animals and other organisms
☒	☐ Clinical data
☒	☐ Dual use research of concern
☒	☐ Plants

Methods

n/a	Involved in the study
☒	☐ ChIP-seq
☒	☐ Flow cytometry
☒	☐ MRI-based neuroimaging

Antibodies

Antibodies used	Mouse monoclonal anti-GAPDH was from Fujifilm WAKO (016-25523). Rabbit anti-GDE5 was produced in house (JBC, 2010).
Validation	All the antibodies used in the study were tested for their reactivity and specificity prior to the use in the study, but not further validation unless noted.

Animals and other research organisms

Policy information about studies involving animals; ARRIVE guidelines recommended for reporting animal research, and Sex and Gender in Research

Laboratory animals	C57BL/6J mice (Charles River, Japan) were used in this study. Gde5+/− mouse was reported in our previous paper (Nakagawa et al., 2016). Gde5-floxed (Gde5flox/flox) mouse was prepared in this paper. Mouse: HSA–Cre transgenic mouse was established by Miniou et al (Nucleic Acids Res, 1999). Duchenne muscular dystrophy mouse models Mdx was provided from Drs. Jun Tanihata (The Jikei University School of Medicine) and Shin'ichi Takeda (National Institute of Neuroscience, National Center for Neurology and Psychiatry) .
Wild animals	n/a
Reporting on sex	Male mice were used in the key experiments, lipid and metabolites analyses, gene expression analysis and contraction force analysis in this study.
Field-collected samples	n/a
Ethics oversight	All animal experiments were conducted in accordance with the animal care protocol approved by the Animal Use Committee of the Hiroshima University (Ethical approval No. C22-30).

Note that full information on the approval of the study protocol must also be provided in the manuscript.
